# Cytotoxic and Pro-Apoptotic Effects of Cassane Diterpenoids from the Seeds of *Caesalpinia sappan* in Cancer Cells

**DOI:** 10.3390/molecules21060791

**Published:** 2016-06-18

**Authors:** Han Bao, Le-Le Zhang, Qian-Yu Liu, Lu Feng, Yang Ye, Jin-Jian Lu, Li-Gen Lin

**Affiliations:** 1 State Key Laboratory of Quality Research in Chinese Medicine, Institute of Chinese Medical Sciences, University of Macau, Macau 999078, China; mb45828@umac.mo (H.B.); lele443119328@126.com (L.-L.Z.); cindy21915@foxmail.com (Q.-Y.L.); 2 Department of Natural Products Chemistry, Shanghai Institute of Materia Medica, Chinese Academy of Sciences, Shanghai 201203, China; fenglu0228@163.com (L.F.); yye@mail.shcnc.ac.cn (Y.Y.)

**Keywords:** *Caesalpinia sappan*, cassane diterpenoids, cytotoxicity, apoptosis, p53

## Abstract

The chemical study on the seeds of *Caesalpinia sappan* led to the isolation of five new cassane diterpenoids, phanginins R‒T (**1**–**3**) and caesalsappanins M and N (**4** and **5**), together with seven known compounds **6**–**12**. Their structures were elucidated on the basis of NMR and HRESIMS analyses. The absolute configurations of compounds **1** and **4** were determined by the corresponding CD spectra. All the isolated compounds were tested for their cytotoxicity against ovarian cancer A2780 and HEY, gastric cancer AGS, and non-small cell lung cancer A549 cells. Compound **1** displayed significant toxicity against the four cell lines with the IC_50_ values of 9.9 ± 1.6 µM, 12.2 ± 6.5 µM, 5.3 ± 1.9 µM, and 12.3 ± 3.1 µM, respectively. Compound **1** induced G1 phase cell cycle arrest in A2780 cells. Furthermore, compound **1** dose-dependently induced A2780 cells apoptosis as evidenced by Hoechst 33342 staining, Annexin V positive cells, the up-regulated cleaved-PARP and the enhanced Bax/Bcl-2 ratio. What’s more, compound **1** also promoted the expression of the tumor suppressor p53 protein. These findings indicate that cassane diterpenoids might have potential as anti-cancer agents, and further *in vivo* animal studies and structural modification investigation are needed.

## 1. Introduction

Cancer is one of the major causes of mortality and death worldwide. The 2014 World Cancer Report confirmed that approximately 14 million people received a new diagnosis of cancer while 8.2 million died in 2012 [1]. Despite the significant advancements made in recent years, treatment of cancer still remains one of the most challenging tasks for human health. Chemotherapy has been recommended as the relatively effective strategy to improve the survival status of patients with ovarian cancer, gastric cancer, lung cancer, *etc.* [2,3,4]. However, serious side effects and acquired drug resistance have become major causes of treatment failure. Therefore, it is imperative to develop new drugs for cancer treatment. The discovery of naturally occurring anticancer agents has been considered as a promising strategy to address this urgent need [5]. Several classes of natural products, including diterpenoids, flavonoids and alkaloids, showed anti-proliferation property by targeting multiple cellular signaling pathways, which have attracted substantial research interests in recent years [6].

*Caesalpinia sappan* Linn. (Fabaceae) is a species of shrubby tree commonly distributed in Southeast Asia and southern China. The heartwood has been used in folk medicines as a blood tonic, expectorant, and has exhibited a wide range of activities, including anti-inflammation [7], anti-influenza virus [8], anti-allergy [9], anti-oxidation [10] and immunomodulation [11]. Previous phytochemical investigations on the seeds of *C. sappan* have led to isolation of a series of cassane-type diterpenoids with potent cytotoxic effect [12,13,14,15,16,17].

In systematic searching for cytotoxic agents, the chloroform-soluble fraction from the ethanol extract of the seeds of *C. sappan* was chemically investigated, resulting in the isolation of twelve cassane diterpenoids, including five new ones, phanginins R–T (**1**–**3**) and caesalsappanins M and N (**4** and **5**), and seven known ones, tomocin C (**6**), phanginin I (**7**), phanginin A (**8**), phanginin F (**9**), caesalpinilinn (**10**), phanginin H (**11**) and caesalsappanin G (**12**). The cytotoxicity of all the diterpenoids was tested on ovarian cancer A2780 and HEY, gastric cancer AGS, and non-small cell lung cancer A549 cells. The pro-apoptotic property of compound **1** was further investigated.

## 2. Results and Discussion

**Figure 1:**
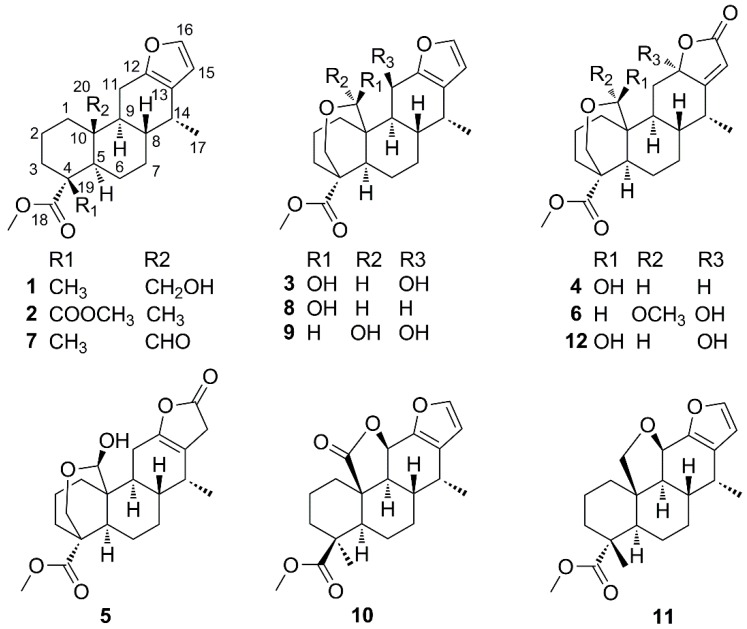
Chemical structures of compounds **1**–**12**.

The chloroform-soluble fraction of the 80% ethanol extract of *C. sappan* was purified by column chromatography over silica gel, MCI gel and preparative HPLC to afford twelve cassane diterpenoids, including five new (compounds **1**–**5**) and seven known ones (compounds **6**–**12**) (Figure 1). The known diterpenoids were identified as tomocin C (**6**) [17], phanginin I (**7**) [12], phanginin A (**8**) [12], phanginin F (**9**) [12], caesalpinilinn (**10**) [18], phanginin H (**11**) [12] and caesalsappanin G (**12**) [16] by comparison of their observed and reported spectroscopic and physical data.

### 2.1. Identification of New Compounds

**Figure 2:**
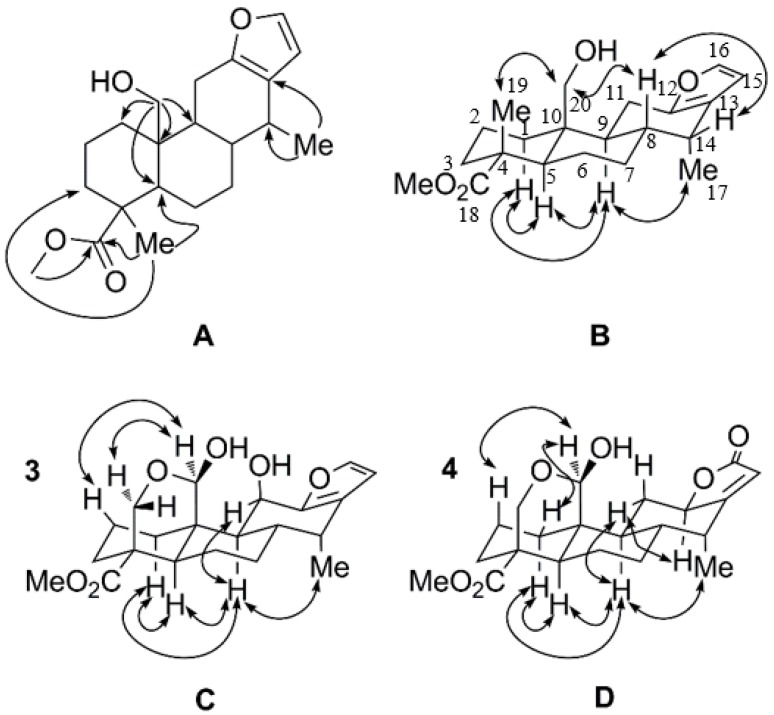
Key HMBC (H→C) (**A**) and ROESY correlations (H↔H) (**B**) for compound **1**. Key ROESY correlations (H↔H) for compounds **3** (**C**) and **4** (**D**).

Phanginin R (**1**) was obtained as a white amorphous powder. The molecular formula, C_21_H_30_O_4_, of **1** was inferred from its HRESIMS spectrum (*m*/*z* 345.2073 [M − H]^−^), with seven degrees of unsaturation. Its IR spectrum showed a broad absorption at 3424 and three sharp absorptions at 1726, 1254 and 1101 cm^−1^, indicating the presences of hydroxy and carboxylic ester groups. The ^1^H-NMR spectrum of **1** exhibited the signals of two aromatic protons at δ_H_ 6.18 (1H, d, *J* = 1.7 Hz, H-15) and 7.21 (1H, d, *J* = 1.7 Hz, H-16) (Table 1), whereas the ^13^C-NMR spectrum showed four sp^2^ carbon signals at δc 149.6 (C-12), 122.1 (C-13), 109.7 (C-15) and 140.3 (C-16) (Table 2), indicating the presence of a furan ring. It was further supported by the maximum UV absorption at 220 nm (log ε 0.58) as well as the IR absorption at 1447 cm^−1^ [19,20]. Additionally, the ^1^H-NMR spectrum of **1** exhibited the signals of one methoxy group (δ_H_ 3.68, s), two methyl groups (δ_H_ 0.97, d, *J* = 7.0 Hz, Me-17; 1.29, s, Me-19) and a hydroxy methyl group (δ_H_ 3.86, d, *J* = 12.1 Hz; 3.97, d, *J* = 12.1 Hz, H-20) (Table 1); and the ^13^C-NMR spectrum indicated the presences of seventeen carbons, including one carbonyl carbon, two quaternary carbons, four methine carbons, seven methylene carbons, two methyl carbons and one methoxy carbon (Table 2). Taken together, compound **1** was deduced to be a cassane-type diterpenoid. After careful comparison, the ^1^H- and ^13^C-NMR data (see Supplementary Materials) of compound **1** were quite similar with those of caesalpinetate [20]. The main differences were the high-field shifts of H-20 (δ_H_ 3.86, 3.97 in compound **1**; δ_H_ 4.15, 4.52 in caesalpinetate) and the absence of an acetyl group, which indicated the acetyl group at C-20 in caesalpinetate might be replaced by a hydroxy group in compound **1**. Next, we took advantage of HMBC experiment to confirm the planar structure of compound **1** (Figure 2A). The HMBC cross-peaks between the singlet methyl group (δ_H_ 1.29) and the carbonyl carbon (δ_C_ 179.2), C-3 (δ_C_ 35.4) and C-5 (δ_C_ 51.5) assigned it as Me-19. The HMBC correlations between the oxygen-bearing protons and C-1 (δ_C_ 31.4), C-5 (δ_C_ 51.5), C-9 (δ_C_ 45.0) and C-10 (δ_C_ 40.6) suggested they were located at C-20. Thus, the planar structure of compound **1** was determined.

The relative configuration of compound **1** was established by analyses of ROESY data (Figure 2B). The NOE correlations between H-9 (δ_H_ 1.61, m) and H-1α (δ_H_ 1.13, m), H-5 (δ_H_ 1.81, m) and Me-17 (δ_H_ 0.97, d, *J* = 7.0 Hz), between H-1α and H-3α (δ_H_ 1.77, m) and H-5, indicated all these protons and Me-17 were axial and α-oriented. Additionally, the cross-peaks between H-8 (δ_H_ 1.88, m) and H-14 (δ_H_ 2.61, m) and H-20 suggested the hydroxymethyl group and these protons were β-oriented. And the NOE signal between the singlet methyl group and H-20 indicated it was β-oriented. The *trans*/*anti*/*trans* system of the three six-membered rings and the orientations of H-8, H-9, M-17 and C-20 in compound **1** were in agreement with those of cassane-type diterpenoids reported from this species previously [12,13,14,15,16,17]. The absolute configuration of compound **1** was further determined by CD spectrum. Based on the previous reports, the cotton effect of cassane-type diterpenoids was mainly effected by the chirality on C-14 [20,21]. Compound **1** exhibited the positive Cotton effect at 216 nm (Δε +76.6) associated with a π–π* transition of the furan chromophore, which indicated the configuration on C-14 as *R*. Thus, the absolute configuration of compound **1** was determined as depicted.

Phanginin S (**2**) was obtained as a white amorphous powder. The HRESIMS of **2** indicated the molecular formula as C_22_H_30_O_5_ (*m*/*z* 397.1990 [M + Na]^+^). The IR spectrum of **2** indicated the presence of carboxylic ester groups (1728, 1260 and 1121 cm^−1^). The ^1^H- and ^13^C-NMR spectra of **2** (Table 1 and Table 2, also see Supplementary Materials) were very similar to those of caesaljapin methyl ester [20]. In the HMBC spectrum, the correlations from the singlet methyl group (δ_H_ 0.74, s) to C-1 (δ_C_ 39.1), C-5 (δ_C_ 50.5), C-9 (δ_C_ 45.2) and C-10 (δ_C_ 37.0) assigned it as Me-20. Two methoxycarbonyl groups were attached to C-4 based on the HMBC correlations from H-3β (δ_H_ 2.42, dd, *J* = 13.0, 1.3 Hz), H-3α (δ_H_ 1.63, m) and H-5 (δ_H_ 2.01, dd, *J* = 12.1, 2.3 Hz) to the carbonyl carbons (δ_C_ 172.6; 173.8). All other HMBC correlations further supported the structure of **2**. Next, the relative configuration of **2** was established by analyses of its ROESY spectrum, which was consistent with reported cassane diterpenoids.

The molecular formula of phanginin T (**3**) was established as C_21_H_28_O_6_ according to the ion peak at *m*/*z* 399.1789 ([M + Na]^+^) in its HRESIMS. In the IR spectrum, the strong absorption at 3435 cm^−1^ indicated the presence of hydroxy groups, and the absorptions at 1722, 1239 and 1144 cm^−1^ suggested the presence of carboxylic ester groups. The ^1^H- and ^13^C-NMR spectra of **3** (Table 1 and Table 2, also see Supplementary Materials) were quite similar to those of phanginin F [20] except for the chemical shifts of H-11, H-19, H-20, C-19 and C-20, indicating compound **3** might have different configuration on the hemiacetal carbon (C-20) with the latter. The relative configuration of compound **3** was established by analyses of the ROESY spectrum (Figure 2C). The cross-peak between H-11 (δ_H_ 4.73, d, *J* = 3.1 Hz) and H-9 (δ_H_ 1.83, m) indicated the hydroxy group at C-11 was β-oriented, which was further supported with the small *J* value between H-9 and H-11 (3.1 Hz). H-20 (δ_H_ 4.98, s) displayed cross-peaks with H-19α (δ_H_ 3.50, d, *J* = 12.6 Hz) and H-2β (δ_H_ 2.01, m), indicating that this proton was *α*-oriented. Accordingly, the structure of compound **3** was established as indicated.

Caesalsappanin M (**4**) was obtained as a white amorphous powder. The HRESIMS of **4**, exhibiting the ion peak at *m*/*z* 399.1787 [M + Na]^+^, established the molecular formula as C_21_H_28_O_6_. The UV and IR spectra showed absorptions for a hydroxy group (3436 cm^−1^) and an α,β-unsaturated butenolide moiety (210 nm; 1729 cm^−1^) [16,22]. The olefinic proton signal at δ_H_ 5.67 (s, H-15) and downfield carbon signals at δc 79.6 (C-12), 175.5 (C-13), 110.9 (C-15) and 174.0 (C-16) in the NMR spectra further supported the presence of the α,β-unsaturated butenolide moiety (Table 1 and Table 2). Besides, the ^1^H-NMR spectrum of **4** showed the signals of a methyl group (δ_H_ 1.04, d, *J* = 7.1 Hz, Me-17), a methoxy group (δ_H_ 3.67, s), an oxygenated methylene group (δ_H_ 3.69, d, *J* = 12.0 Hz, H-19α; 4.33, dd, *J* = 11.9, 2.1 Hz, H-19β) and two oxygenated methine groups (δ_H_ 4.83, d, *J* = 2.1 Hz, H-20; 4.83, dd, *J* = 11.6, 6.0 Hz, H-12) (Table 1). Except for the methoxy group (δc 51.8) and the α,β-unsaturated butenolide moiety, the ^13^C-NMR and DEPT spectra of **4** disclosed sixteen carbon signals, corresponding to one methyl carbon, seven methylene carbons, five methine carbons, two quaternary carbons and one carbonyl carbon (Table 2). The NMR data of **4** (see Supplementary Materials) quite resembled to those of caesalsappanin G [16]. The major difference was the oxygenated methine (δ_H_ 4.83; δc 79.6) in compound **4** instead of the hemiketal carbon (δ_C_ 105.9) in caesalsappanin G, indicating a hydrogen atom in **4** might replace the hydroxyl group at C-12 in caesalsappanin G. To determine the structure of **4**, the HMBC and HSQC experiments were carried out. The HMBC cross-peaks of the oxymethine proton (δ_H_ 4.83) with C-11 (δc 33.8), C-13 (δc 175.5), C-14 (δc 36.9) and C-15 (δc 110.9) suggested it at C-12. The relative configuration of **4** was determined by the ROESY experiment (Figure 2D). The key NOE correlations between H-20 and H-1β (δ_H_ 2.05, m) and H-2β (δ_H_ 2.28, m) suggested this proton was α-oriented. Moreover, the NOE cross-peak of H-12/H-11α (δ_H_ 2.60, m) indicated H-12 was also α-oriented. Among the cassane diterpenoids isolated from the genus *Caesalpinia*, about twenty compounds contain an α,β-unsaturated butenolide moiety fused on ring C, all of which possess the same configuration at C-12 [16,23,24,25]. The substituents at C-12 could be a hydrogen atom, hydroxy, methoxy, ethoxy and acetyl group. From biogenetic consideration, the configuration of **4** was consistent with those diterpenoids reported previously. The absolute configuration of **4** was deduced from the CD curve of its γ-lactone chromophore. The negative π–π* Cotton effect at 228 nm (Δε-9.6) indicated an *S* configuration at C-12 [17,25], thus confirming the proposed structure of compound **4**.

Caesalsappanin N (**5**) was obtained as a white amorphous powder and showed a quasi-molecular ion peak at *m*/*z* 399.1780 [M + Na]^+^ in the HRESIMS, corresponding to the formula C_21_H_28_O_6_. The IR spectrum of **5** indicated the presence of hydroxy groups (3344 cm^−1^). The NMR data of **5** (Table 1 and Table 2, also see Supplementary Materials) were nearly identical to those of caesalsappanin G [16]. Carefully comparison revealed that a methylene (δ_H_ 3.18, d, *J* = 18.6 Hz, H-15α; 3.01, d, *J* = 18.6 Hz, H-15β; δ_C_ 34.8, C-15) and a tetrasubstituted alkene (δ_C_ 149.1, C-12; 115.6, C-13) in **5** instead of a hemiketal group (δ_C_ 105.9) and a trisubstituted alkene (δ_H_ 5.68, s; δ_C_ 173.6, 113.6) in caesalsappanin G. It suggested that the α,β-unsaturated butenolide moiety in caesalsappanin G might be replaced by a β,γ-unsaturated butenolide structure in **5**, which was supported by the lower UV absorption wavelength (205 nm). In the HMBC spectrum, the correlations between the geminal protons (δ_H_ 3.18, 3.01) and C-14 (δ_C_ 32.2), C-16 (δ_C_ 176.9) and C-17 (δ_C_ 14.3) indicated them as H-15. The cross-peaks of the olefinic carbon at δ_C_ 149.1 with H-11 (δ_H_ 2.25, 2.36, m), H-14 (δ_H_ 2.38, m) and H-15, and the olefinic carbon at δ_C_ 115.6 with H-11, H-14, H-15 and C-17 (δ_H_ 0.88, d, *J* = 7.0 Hz), assigned them as C-12 and C-13, respectively. The relative configuration of compound **5** was consistent with that of caesalsappanin G, indicating by the ROESY experiment, thus conforming the structure of **5** as indicated.

### 2.2. Cytotoxicity Assay

To date a series of cassane-type diterpenoids have been isolated from *C sappan*, and some of them were demonstrated to possess cytotoxic effects against several cancer cell lines [26]. In a chemical study of the seeds of *C. sappan*, 11 cassane-type diterpenoids, phanginins A–K, were identified. Among them, phagninin I (**7**) showed moderate inhibitory activity against KB cell line with the IC_50_ value of 12.8 μM [12]. In another study, phanginins D, I (**7**) and H (**11**) from the seeds of Vietnamese *C. sappan* were reported to show effective inhibition against leukemic HL60 cells with the IC_50_ values of 11.7  ±  1.6, 16.4  ±  1.5 and 22.5  ±  5.1 μM, respectively [15]. Besides, caesalsappanin J from the seeds of *C. sappan* exhibited relative strong anti-proliferative activity against KB cancer cells with the IC_50_ value of 7.4 μM [16]. Phanginins L, N, O, and P from the seeds of *C. sappan* showed weak cytotoxicity against three human cancer cell lines HepG-2, MCF-7 and HCT-8 (IC_50_ > 20 μM) [13,14]. In a recent study of the seed kernels of Vietnamese *C. sappan*, tomocin A, phanginins A, F, and H were found to exhibit mild preferential cytotoxicity against PANC-1 human pancreatic cancer cells under nutrition-deprived condition but not in normal nutrient-rich conditions [17].

**Figure 3:**
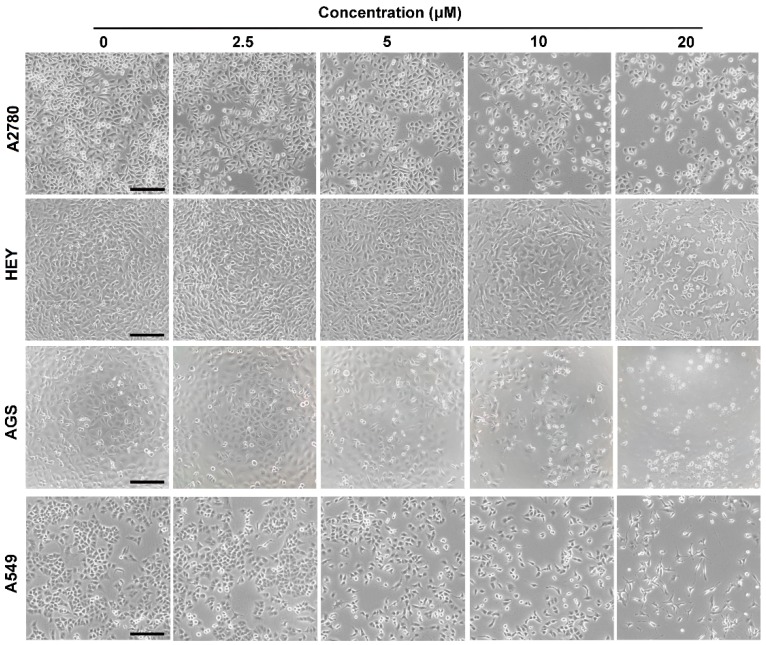
Morphology changes of A2780, HEY, AGS and A549 cells after treated with series concentrations of compound **1** (2.5 to 20 μM). Bar: 200 μm.

Herein, the human ovarian cancer A2780 and HEY, gastric cancer AGS, and non-small cell lung cancer A549 cells were used to evaluate the cytotoxicity of the compounds **1**–**12**. As shown in Table 3, compounds **2**–**6**, **11** and **12** didn’t show obvious effect on the cell lines up to 20 μM. Compounds **1**, **7** and **8** exhibited relative higher toxicity on A2780 cells while compounds **1** and **8** showed higher potential on HEY, AGS and A549 cells. Herein, paclitaxel was used as the positive control, and viabilities of A2780, HEY, AGS and A549 cells after treated with paclitaxel (250 nM) were 50.19% ± 5.27%, 36.25% ± 5.82%, 55.43% ± 4.58% and 33.28% ± 8.81%, respectively. After treatment with compound **1**, morphology changes of A2780, HEY, AGS and A549 cells were observed as becoming slender or suspended (Figure 3). The IC_50_ values of compound **1** on A2780, HEY, AGS and A549 cells were 9.9 ± 1.6 μM, 12.2 ± 6.5 μM, 5.3 ± 1.9 μM, and 12.3 ± 3.1 μM, respectively, indicated that compound **1** exhibits cytotoxicity on the four cancer cell lines. Based on the results, the presence of a hydroxy (**1** and **8**) or an aldehyde group (**7**) at C-20 is important for the cytotoxicity of cassane diterpenoids; while the existence of a hydroxy group at C-11 (**3** and **9**) attenuates the cytotoxicity.

### 2.3. Compound ***1*** Mediates G1 Cell Cycle Arrest

**Figure 4:**
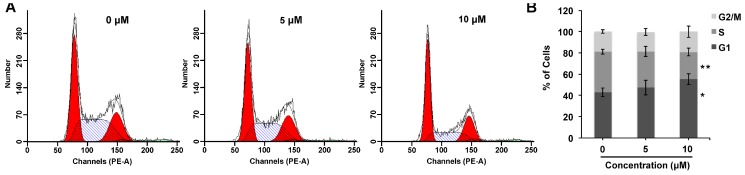
Compound **1** induced G1 phase cell cycle arrest in ovarian cancer cells. (**A**) A2780 cells were treated with indicated concentrations of compound **1** for 24 h. Cell cycle assays were conducted using flow cytometry; (**B**) The fractions of cells at the G1, S and G2/M phases were semi-quantified. * *p* < 0.05 and ** *p* < 0.01, compared with the 0 μM compound **1** treatment (control).

Cell cycle arrest is a critical aspect of the anti-proliferative activity. Analysis of the distribution of cell cycle showed that compound **1** induced G1 phase cell cycle arrest in ovarian cancer A2780 cells at low concentrations. As shown in Figure 4, after 24 h exposure to relative concentrations of compound **1**, the fraction of A2780 cells at G1 phase increased from 42.99% ± 4.14% to 55.37% ± 5.23% (10 μM), while the cells at S phase decreased from 38.07% ± 2.37% to 25.47% ± 3.57% (10 μM), respectively. Thus, the cytotoxicity of compound **1** might partially in consequence of the G1 phase cell cycle arrest.

### 2.4. Pro-apoptotic and p53 Suppressing Activities of Compound ***1***

Currently, the cytotoxicity mechanism(s) of cassane-type diterpenoids have scarcely been studied. Phanginin D was reported to induce apoptosis in HL-60 cells as evidenced by increment of caspase-3 activity and cleavage of procasepase-3 and PARP [15].

**Figure 5:**
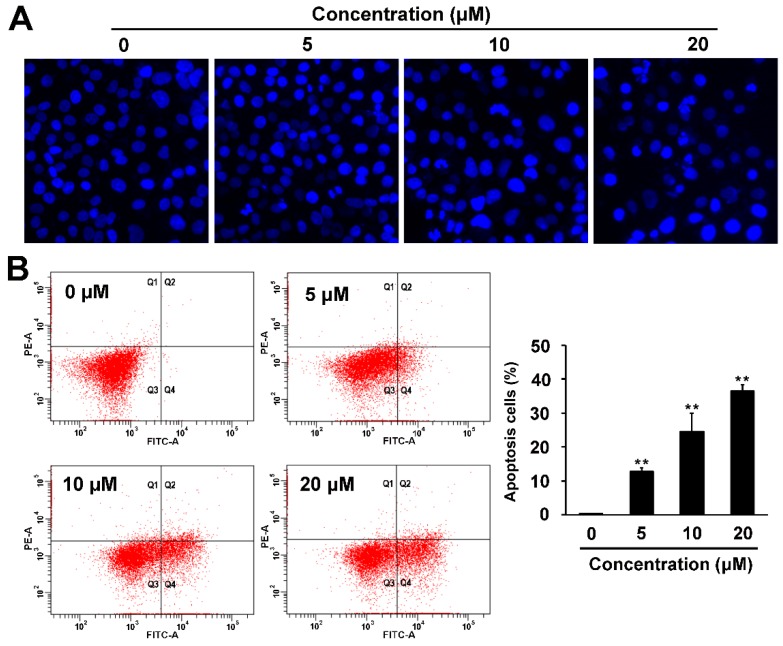

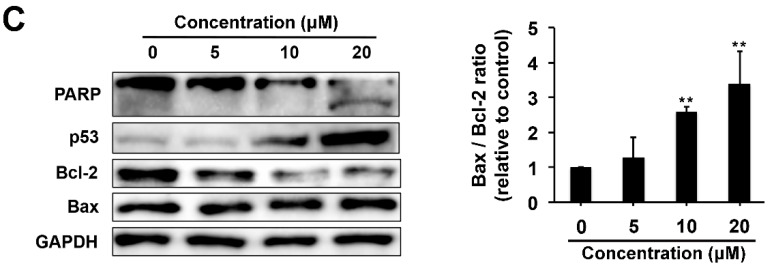
Compound **1** induced apoptosis in ovarian cancer cells. A2780 cells were treated with indicated concentrations of compound **1** for 24 h. (**A**) Apoptotic body of A2780 cells were detected by Hoechst 33342 staining; (**B**) Percentage of apoptotic cells was analyzed with Annexin V/PI assay; (**C**) Protein levels of PARP, p53, Bcl-2 and Bax were determined by western blot analysis. The relative Bax/Bcl-2 ratio was calculated. ** *p* < 0.01, compared with the 0 μM compound **1** treatment (control).

As induction of apoptosis is one of the major causes that mediate cytotoxicity, we conducted the Hoechst 33342 staining, Annexin V/PI double staining and western blot analysis to investigate whether compound **1** induced apoptosis on ovarian cancer A2780 cells. After treatment with compound **1** for 24 h, nuclei of some A2780 cells began to shrink into semilune-shape at concentration of 5 μM and apoptotic bodies appealed at concentrations of 10 and 20 μM (Figure 5A). Annexin V/PI double staining analysis showed that the number of apoptotic cells increased significantly in a concentration dependent manner. 36.5% ± 1.8% cells became Annexin V positive after treatment with 20 μM of compound **1** (Figure 5B).

The expression levels of several apoptosis related proteins were further detected. Cleavage of PARP is a sensitive parameter of apoptosis. After compound **1** treatment, the cleavage fragment of PARP was obviously up-regulated (Figure 5C), which is consistent with the enhanced apoptosis. The Bcl-2 protein family has been reported to play a key role in cell apoptosis [27]. Bcl-2 is an anti-apoptosis protein that could inhibit cell apoptosis, and Bax is a homologue of Bcl-2 that could promote apoptosis. The Bax/Bcl-2 ratio determines the susceptibility of cells to apoptosis. We found that compound **1** could obviously increase the Bax/Bcl-2 ratio.

Besides, the major tumor suppressor protein p53 is one of the most frequently mutated tumor suppressors identified so far in human cancers, and stable expression of p53 is crucial for its tumor suppressor function [28,29]. Our results showed that compound 1 could obviously up-regulate the expression level of p53 (Figure 5C), which further supported compound **1** to be a candidate for cancer treatment.

## 3. Materials and Methods

### 3.1. General Procedures

Optical rotation data were obtained using an Autopol VI polarimeter (DKSH Pharmaceutical Ltd., Shanghai, China). UV data were recorded with a CARY 50 spectrophotometer (Varian, Seattle, WA, USA). CD data were recorded with a J-810 circular dichroism spectrophotometer (JASCO, Easton, MD, USA). IR spectra were recorded on a Spectrum-100 FTIR spectrometer (Perkin Elmer, Waltham, MA, USA) using KBr disks. NMR spectra were recorded on an ASCEND 600 MHz/54 mm-NMR spectrometer (Bruker, Beijing, China). The chemical shift (δ) values were given in ppm with TMS as internal standard, and coupling constants (*J*) were in Hz. ESIMS and HRESIMS spectra were recorded on an LTQ-Orbitrap XL spectrometer (Thermo Fisher Scientific, Waltham, MA, USA). All solvents were analytical grade (Tianjin Chemical Plant, Tianjin, China). Silica gel used for flash chromatography and precoated silica gel GF254 plates used for TLC were produced by Qingdao Haiyang Chemical Co., Ltd. (Qingdao, China). TLC spots were viewed at 254 nm and visualized by spraying with 10% sulfuric acid in alcohol. MCI gel (CHP20P, 75–150 μm, Mitsubishi Chemical Industries Ltd., Tokyo, Japan) was used for column chromatography (CC). Preparative HPLC was performed on a LC-20AP instrument (Shimadzu, Tokyo, Japan) with a SPD-M20A PDA detector. Chromatographic separation was carried out on a C18 column (19 mm × 250 mm, 5 μm, SunFire™, Waters, Milford, MA, USA), using a gradient solvent system comprised of H_2_O (A) and MeCN (B) at a flow rate of 10 mL/min.

### 3.2. Plant Material

The seeds of *C. sappan* Linn. (15.1 kg) were collected in Guangxi, People’s Republic of China, and identified by Professor Jin-Gui Shen from Shanghai Institute of Materia Media, Chinese Academy of Sciences. A voucher was deposited at the herbarium of Institute of Chinese Medical Sciences, University of Macau (LL-20140601).

### 3.3. Extraction and Isolation

The air-dried seeds of *C. sappan* were ground into powder and extracted with petroleum ether three times to remove lipids. Then the residues were extracted three times with 40 L 80% ethanol at room temperature (each 2 days). After evaporation of the collected percolate, the crude extract (500.4 g) was suspended in 2.0 L H_2_O and extracted with chloroform (1.5 L × 3), ethyl acetate (1.5 L × 3) and *n*-butanol (1.5 L × 3), successively. The chloroform extract (370.0 g) was subjected to chromatography over silica gel and eluted with petroleum ether–acetone (19:1 to 1:1, *v*/*v*), to yield ten major fractions (C1 to C10). Phanginin A (**8**) (2.1 g) was crystalized from fraction C6. Fractions C1 and C5 were subjected to MCI gel column chromatography eluting with H_2_O/MeOH (4:6 to 0:1, *v*/*v*) to yield subfractions C1A‒C1J and C5A‒C5K, respectively. C1D was further separated by preparative HPLC eluted with gradient H_2_O/MeCN (2:3 to 0:1, *v*/*v*) to obtain phanginin H (**11**) (10.1 mg) and caesalpinilinn (**10**) (3.4 mg). Phanginin I (**7**) (516.8 mg) was crystalized from subfraction C1F. C1H was applied to a silica gel column and eluted with petroleum ether/ethyl acetate (30:1 to 0:1 *v*/*v*) to obtain phanginin R (**2**) (1.4 mg). Fraction C5C was subjected to preparative HPLC eluted with gradient H_2_O/MeCN (1:1 to 0:1, *v*/*v*) to obtain phanginin Q (**1**) (2.5 mg), caesalsappanin M (**4**) (1.3 mg), caesalsappanin N (**5**) (1.1 mg), tomocin C (**6**) (1.8 mg) and caesalsappanin G (**12**) (3.4 mg). C5I was subjected to a silica gel column eluted with petroleum ether/ethyl acetate (25:1 to 0:1 *v*/*v*), to yield a series of subfractions (C5I1 to C5I9). Phanginin S (**3**) (10.0 mg) and phanginin F (**9**) (14.4 mg) were obtained from C5I4 through preparative HPLC eluted with gradient H_2_O/MeCN (9:11 to 0:1, *v*/*v*).

### 3.4. Spectroscopic Data

Phanginin R (**1**). White amorphous powder; ${\left[\alpha \right]}_{D}^{20}$ +61.4 (*c* 0.03, MeOH); UV (MeOH) λ_max_ (log ε) 220.0 (0.58) nm; CD (MeOH, nm) λ_max_ (Δ*ε*) 193 (−31.1), 216 (+76.6); IR ν_max_ (KBr) 3461 (strong, broad), 2926, 2866, 1726, 1447, 1254, 1101, 1045, 899 cm^−1^; ^1^H- and ^13^C-NMR data see Table 1 and Table 2; HRSEIMS *m*/*z* 345.2073 [M − H]^−^ (calcd for C_21_H_29_O_4_, 345.2065).

Phanginin S (**2**). White amorphous powder; ${\left[\alpha \right]}_{D}^{20}$ +5.8 (*c* 0.01, MeOH); UV (MeOH) λ_max_ (log ε) 205.0 (2.15) nm; IR ν_max_ (KBr) 2924, 2854, 1728, 1636, 1460, 1384, 1260, 1121, 1078 cm^−1^; ^1^H- and ^13^C-NMR data see Table 1 and Table 2; HRSEIMS *m*/*z* 397.1990 [M + Na]^+^ (calcd for C_22_H_30_O_5_Na, 397.1991).

Phanginin T (**3**). White amorphous powder; ${\left[\alpha \right]}_{D}^{20}$ +9.2 (*c* 0.03, MeOH); UV (MeOH) λ_max_ (log ε) 220.0 (0.70) nm; IR ν_max_ (KBr) 3435 (strong, broad), 2937, 2866, 1722, 1458, 1239, 1144, 1071 cm^−1^; ^1^H- and ^13^C-NMR data see Table 1 and Table 2; HRSEIMS *m*/*z* 399.1789 [M + Na]^+^ (calcd for C_21_H_28_O_6_Na, 399.1784).

Caesalsappanin M (**4**). White amorphous powder; ${\left[\alpha \right]}_{D}^{20}$ −56.3 (*c* 0.01, MeOH); UV (MeOH) λ_max_ (log ε) 210.1 (0.56) nm; CD (MeOH, nm) λ_max_ (Δε) 196 (+7.2), 228 (−9.6); IR ν_max_ (KBr) 3436 (strong, broad), 2923, 2852, 1729, 1641, 1460, 1384, 1260, 1098, 1040 cm^−1^; ^1^H- and ^13^C-NMR data see Table 1 and Table 2; HRSEIMS *m*/*z* 399.1787 [M + Na]^+^ (calcd for C_21_H_28_O_6_Na, 399.1784).

Caesalsappanin N (**5**). White amorphous powder; ${\left[\alpha \right]}_{D}^{20}$ −0.8 (*c* 0.01, MeOH); UV (MeOH) λ_max_ (log ε) 205.0 (0.77) nm; IR ν_max_ (KBr) 3344 (strong, broad), 2927, 2866, 1726, 1458, 1262, 1099, 1039 cm^−1^; ^1^H- and ^13^C-NMR data see Table 1 and Table 2; HRSEIMS *m*/*z* 399.1780 [M + Na]^+^ (calcd for C_21_H_28_O_6_Na, 399.1784).

### 3.5. Reagents

The compounds were dissolved in dimethyl sulfoxide (DMSO, Sigma-Aldrich Co., St. Louis, MO, USA) as a stock solution and stored at −20 °C. Dulbecco’s modified Eagle’s medium (DMEM), Ham’s F-12K (Kaighn’s) medium, RPMI 1640 medium, 0.25% trypsin-EDTA, fetal bovine serum (FBS), penicillin-streptomycin (10,000 units/mL of penicillin and 10,000 μg/mL of streptomycin) and phosphate-buffered saline (PBS) were purchased from Gibco (Carlsbad, CA, USA). Paclitaxel and 3-(4,5-Dimethyl-2-thiazolyl)-2,5-diphenyltetrazolium bromide (MTT) were purchased from Sigma (Saint Louis, MO, USA). Hoechst 33342 was obtained from Molecular Probes (Grand Island, NY, USA). Primary antibodies against PARP, p53, Bcl-2, Bax, and GAPDH, together with the secondary antibodies, were obtained from Cell Signaling Technology, Inc. (Beverly, MA, USA).

### 3.6. Cell Culture

The human ovarian cancer A2780 cells were acquired from KeyGEN Biotech (Nanjing, Jiangsu, China), HEY cells were kindly provided by Dr. Wen-An Qiang (Northwestern University Feinberg School of Medicine, Chicago, IL, USA). A2780 and HEY were cultured in DMEM medium supplemented with 10% (*v*/*v*) FBS and 1% (*v*/*v*) penicillin-streptomycin. Human gastric cancer AGS cells were obtained from cell bank of Chinese Academy of Sciences (Shanghai, China) and cultured in Ham’s F-12K (Kaighn’s) medium supplemented with 10% (*v*/*v*) FBS and 1% (*v*/*v*) Penicillin-Streptomycin. Human non-small cell lung cancer A549 cells were obtained from American Type Culture Collection (Rockville, MD, USA) and cultured in RPMI 1640 medium containing 10% (*v*/*v*) FBS and 1% (*v*/*v*) penicillin-streptomycin. Cells were grown in a standard humidified incubator with 5% CO_2_ at 37 °C.

### 3.7. MTT Assay

Viability of the cells after treatment with the pure compounds was determined by MTT assay. Exponentially growing A2780, HEY, AGS and A549 cells were seeded into 96-well plates. Upon reaching approximately 70%−80%, the cells were treated with series concentrations of different compounds for 48 h. Paclitaxel was used as a positive control. After treatment, 1 mg/mL MTT solution was added to each well and the 96-well plates were further incubated for 4 h at 37 °C. 100 μL of DMSO was added to each well to dissolve the needle-like formazan crystals formed by viable cells. Absorbance at 570 nm was measured by a microplate reader (1420 Multilabel Counter Victor 3, Perkin Elmer, Wellesley, MA, USA).

### 3.8. Cell Cycle Analysis

A2780 cells were seeded into 6-well plates and cultured overnight. After 24 h treatment, cells were trypsinized, washed with PBS and harvested by centrifugation. Then, cells were resuspended in cold ethanol (70%) and fixed overnight at 4 °C. After washed with PBS, cells were incubated with PI solution (20 μg/mL) for 30-min in the dark at room temperature. A total of 10,000 cells were collected and analyzed using flow cytometry (FACS Canto™, BD Bioscience, Franklin Lakes, NJ, USA).

### 3.9. Hoechst 33342 Staining Assay

A2780 cells were seeded into 96-well plates and cultured overnight. After 24 h treatment, cells were washed with PBS and fixed with 4% formaldehyde for 30 min. Then, cells were stained with Hoechst 33342 (1 μg/mL) for 30 min. After PBS washing, fluorescent images of nuclei were captured by In Cell Analyzer 2000 (GE Healthcare, Little Chalfont, UK).

### 3.10. Annexin V/PI Staining Assay

A2780 cells were seeded into 6-well plates and cultured overnight. After 24 h treatment, cells were trypsinized, washed with PBS and harvested by centrifugation. Apoptotic cells were detected using an Annexin V-FITC/PI double staining apoptosis detection kit (Beyotime Biotechnology, Shanghai, China). Briefly, a total of 10,000 cells were collected and analyzed by BD FACS Canto™ flow cytometry (BD Biosciences, San Jose, CA, USA).

### 3.11. Western Blot Analysis

A2780 cells were seeded into 6-well plates and cultured overnight. After 24 h treatment, cells were lysed in the RIPA lysis buffer containing 1% protease inhibitor cocktail and 1% phenylmethane-sulfonylfluoride. Protein concentrations of the lysates were then determined using a BCA^TM^ Protein Assay Kit (Pierce, Rockford, IL, USA). 20 µg of total proteins were separated by sodium dodecyl sulfate-polyacrylamide gel electrophoresis, then transferred to polyvinylidene fluoride membranes and blocked with 5% nonfat milk for 2 h at room temperature. The membranes were probed with specific primary antibodies against PARP, p53, Bcl-2, Bax and GAPDH overnight at 4 °C and then probed with corresponding secondary antibodies for 1 h at room temperature. Then, specific protein bands were visualized using the CheniDoc MP Imaging System, and quatification was performed with Image Lab 5.1. Equal protein loading was verified by probing with anti-GAPDH antibody.

### 3.12. Statistical Analysis

Data were expressed as mean values and standard deviation. Statistical significances were analyzed by one-way analysis of variance using SPSS 17 software (Statistical Package for the Social Sciences, SPSS Inc., Chicago, IL, USA). * *p* < 0.05 and ** *p* < 0.01 were considered as the significant difference.

## 4. Conclusions

Discovery of novel anti-cancer compounds from natural products have received more and more attention owing to the rich source and enormous structural diversity. In the current study, twelve cassane diterpenoids were discovered from the seeds of *C. sappan*, including five new compounds, and compound **1** showed significant cytotoxicity on four cancer cell lines and apoptotic inducing potential against A2780 cells. In summary, these findings indicate that cassane diterpenoids might have potential as anti-cancer agents, and further *in vivo* animal studies and structural modification investigation are needed.

## Figures and Tables

**Table 1:** ^1^H-NMR spectroscopic data (600 MHz, CDCl_3_) for compounds **1**‒**5** (δ_H_ in ppm, *J* in Hz).

Position	1	2	3	4	5
1α	1.13, m	1.14, dd (13.1, 4.8)	1.48, m	1.20, m	1.26, m
1β	2.27, m	1.75, m	1.78, m	2.05, m	1.46, m
2α	1.60, m	1.87, m	1.61, m	1.64, m	1.62, m
2β	1.44, m	1.68, m	2.01, m	2.28, m	2.31, m
3α	1.77, m	1.63, m	1.56, m	1.93, m	1.94, m
3β	1.61, m	2.42, dd (13.0, 1.3)	2.01, m	2.05, m	2.00, m
5	1.81, m	2.01, dd (12.1, 2.3)	1.83, m	1.68, m	1.65, m
6α	1.21, m	1.45, m	1.57, m	1.21, m	1.24, m
6β	1.41, m	1.64, m	1.75, m	2.10, m	1.62, m
7α	1.40, m	1.57, m	1.19, m	1.36, m	1.36, m
7β	1.70, m	1.76, m	1.76, m	1.56, m	1.67, m
8	1.88, m	1.78, m	2.17, m	2.32, m	2.34, m
9	1.61, m	1.56, m	1.83, m	1.50, m	2.24, m
11α	2.71, dd (16.8, 6.4)	2.60, dd (16.9, 6.8)	4.73, d (3.1)	2.60, m	2.25, m
11β	2.34, m	2.35, dd (16.9, 11.8)	-	1.74, m	2.36, m
12	-	-	-	4.83, dd (11.6, 6.0)	-
14	2.61, m	2.62, m	2.66, m	2.92, m	2.38, m
15	6.18, d (1.7)	6.18, d (1.7)	6.23, d (1.7)	5.67, s	α: 3.18, d (18.6) β: 3.01, d (18.6)
16	7.21, d (1.7)	7.22, d (1.7)	7.32, d (1.7)	-	-
17	0.97, d (7.0)	0.99, d (7.1)	0.98, d (7.1)	1.04, d (7.1)	0.88, d (7.0)
19α	1.29, s	-	3.50, d (12.5)	3.69, d (12.0)	3.72, d (11.7)
19β			4.89, d (12.6)	4.33, dd (11.9, 2.1)	4.35, dd (11.8, 2.5)
20	α: 3.86, d (12.1) β: 3.97, d (12.1)	0.74, s	4.98, s	4.83, d (2.1)	4.98, s
18-OMe	3.68, s	3.71, s	3.72, s	3.67, s	3.67, s
19-OMe	-	3.74, s	-	-	-

**Table 2:** ^13^C-NMR spectroscopic data (125 MHz, CDCl_3_) for compounds **1**‒**5** (δ_C_ in ppm).

Position	1	2	3	4	5
1	31.4	39.1	34.9	37.8	37.8
2	17.6	19.1	17.9	21.0	20.9
3	35.4	34.7	35.7	35.7	35.6
4	49.4	57.6	47.3	45.6	45.6
5	51.5	50.5	46.2	45.1	45.1
6	23.5	25.7	27.8	23.9	23.5
7	30.5	31.7	28.1	29.0	21.9
8	36.6	36.1	38.1	41.2	36.5
9	45.0	45.2	45.1	41.7	41.9
10	40.6	37.0	43.4	38.7	38.2
11	23.1	22.5	70.1	33.8	29.2
12	149.6	149.5	146.7	79.6	149.1
13	122.1	122.5	129.5	175.5	115.6
14	31.4	31.7	32.6	36.9	32.2
15	109.7	109.7	109.5	110.9	34.9
16	140.3	140.6	143.1	174.0	176.9
17	16.9	17.8	14.2	13.3	14.3
18	179.2	173.8	176.1	177.2	175.7
19	19.1	172.6	67.1	61.5	61.9
20	61.2	13.7	106.2	96.8	97.3
18-OMe	52.0	52.8	52.2	51.8	51.8
19-OMe		52.0			

**Table 3:** Viability of A2780, HEY, AGS and A549 cells after treated with the twelve compounds (20 μM). Cells were treated with 20 μM of the twelve compounds for 48 h and the cell viability was detected by MTT assay.

Compounds	A2780	HEY	AGS	A549
**1**	10.4% ± 4.7%	10.2% ± 9.8%	4.9% ± 1.3%	32.9% ± 13.0%
**2**	92.0% ± 7.1%	95.7% ± 6.4%	80.8% ± 9.7%	80.0% ± 8.0%
**3**	74.8% ± 8.2%	79.9% ± 12.7%	71.7% ± 18.2%	74.6% ± 5.4%
**4**	97.3% ± 7.4%	95.2% ± 6.0%	80.8% ± 15.7%	79.9% ± 10.0%
**5**	91.7% ± 1.4%	97.2% ± 2.8%	83.0% ± 16.6%	84.9% ± 10.0%
**6**	95.2% ± 1.0%	91.3% ± 9.6%	82.3% ± 9.9%	83.5% ± 7.6%
**7**	37.9% ± 5.6%	68.2% ± 5.5%	69.5% ± 8.9%	73.5% ± 12.0%
**8**	49.5% ± 5.8%	41.6% ± 9.0%	14.6% ± 2.3%	50.9% ± 12.3%
**9**	64.4% ± 4.6%	74.1% ± 7.0%	71.1% ± 12.7%	72.8% ± 4.7%
**10**	57.4% ± 4.5%	71.4% ± 1.9%	62.7% ± 13.5%	75.2% ± 9.8%
**11**	95.4% ± 2.7%	91.7% ± 6.1%	85.8% ± 9.4%	74.8% ± 13.8%
**12**	92.1% ± 6.6%	97.5% ± 2.0%	82.1% ± 14.0%	82.2% ± 10.6%

## Supplementary Materials

The ^1^H- and ^13^C-NMR data of compounds **1**–**5** can be accessed at: http://www.mdpi.com/1420-3049/21/6/791/s1.

## Abbreviations

The following abbreviations are used in this manuscript:

CD	Circular Dichroism
^13^C-NMR	^13^C Nuclear Magnetic Resonance
1D-NMR	1 Dimension Nuclear Magnetic Resonance
2D-NMR	2 Dimension Nuclear Magnetic Resonance
DMSO	Dimethyl Sulphoxide
ESIMS	Electrospray Ionization Mass Spectrometry
FBS	Fetal Bovine Serum
HMBC	Heteronuclear Multiple Bond Correlation
^1^H-NMR	^1^H Nuclear Magnetic Resonance
HRESIMS	High-resolution ESIMS
HSQC	Heteronuclear Single-quantum Correlation
IR	Infrared Spectroscopy
MTT	3-(4,5-Dimethyl-2-thiazolyl)-2,5-diphenyltetrazolium Bromide
PBS	Phosphate-buffered Saline
Preparative HPLC	Preparative High Performance Liquid Chromatography
ROESY	Rotating-frame Nuclear Overhauser Enhancement Spectroscopy
SDS	Sodium Dodecyl Sulfate
TLC	Thin Layer Chromatography
UV	Ultraviolet Spectroscopy
