# Supplementary material for: Cytotoxic and Pro-Apoptotic Effects of Cassane Diterpenoids from the Seeds of Caesalpinia sappan in Cancer Cells
Source: Molecules. 2016 Jun 18;21(6):791. doi: 10.3390/molecules21060791 (PMC6274183; doi:10.3390/molecules21060791)
Supplement: Supplementary file 1 [file molecules-21-00791-s001.pdf]

Supplementary Materials: Cytotoxic and Pro-apoptotic Effects of Cassane Diterpenoids from the Seeds of *Caesalpinia sappan* in Cancer Cells

Han Bao, Le-Le Zhang, Qian-Yu Liu, Lu Feng, Yang Ye, Jin-Jian Lu and Li-Gen Lin

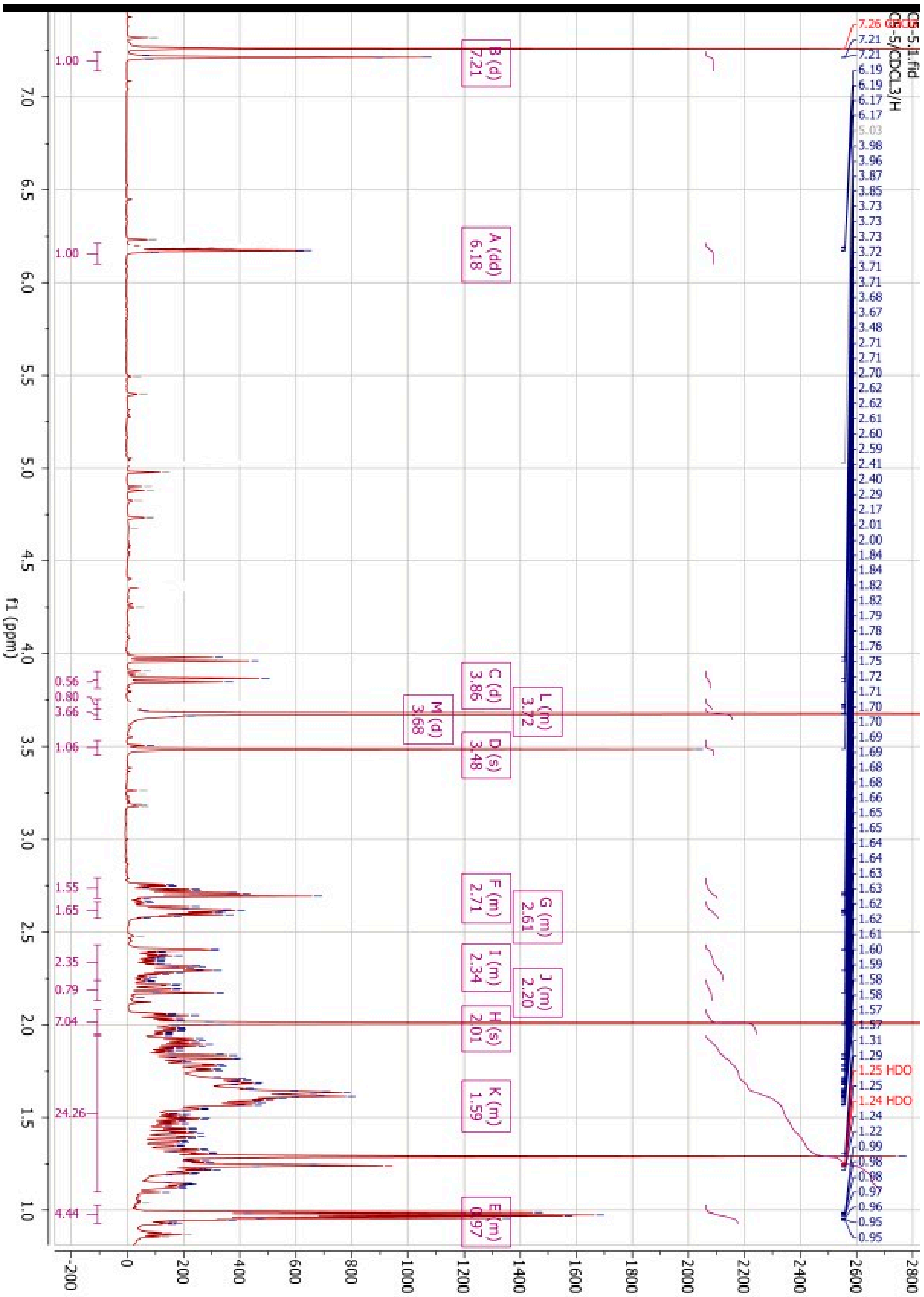

Figure S1. ^{1}H-NMR spectrum (600 MHz, $CDCl_3$) of phanginin R (**1**).

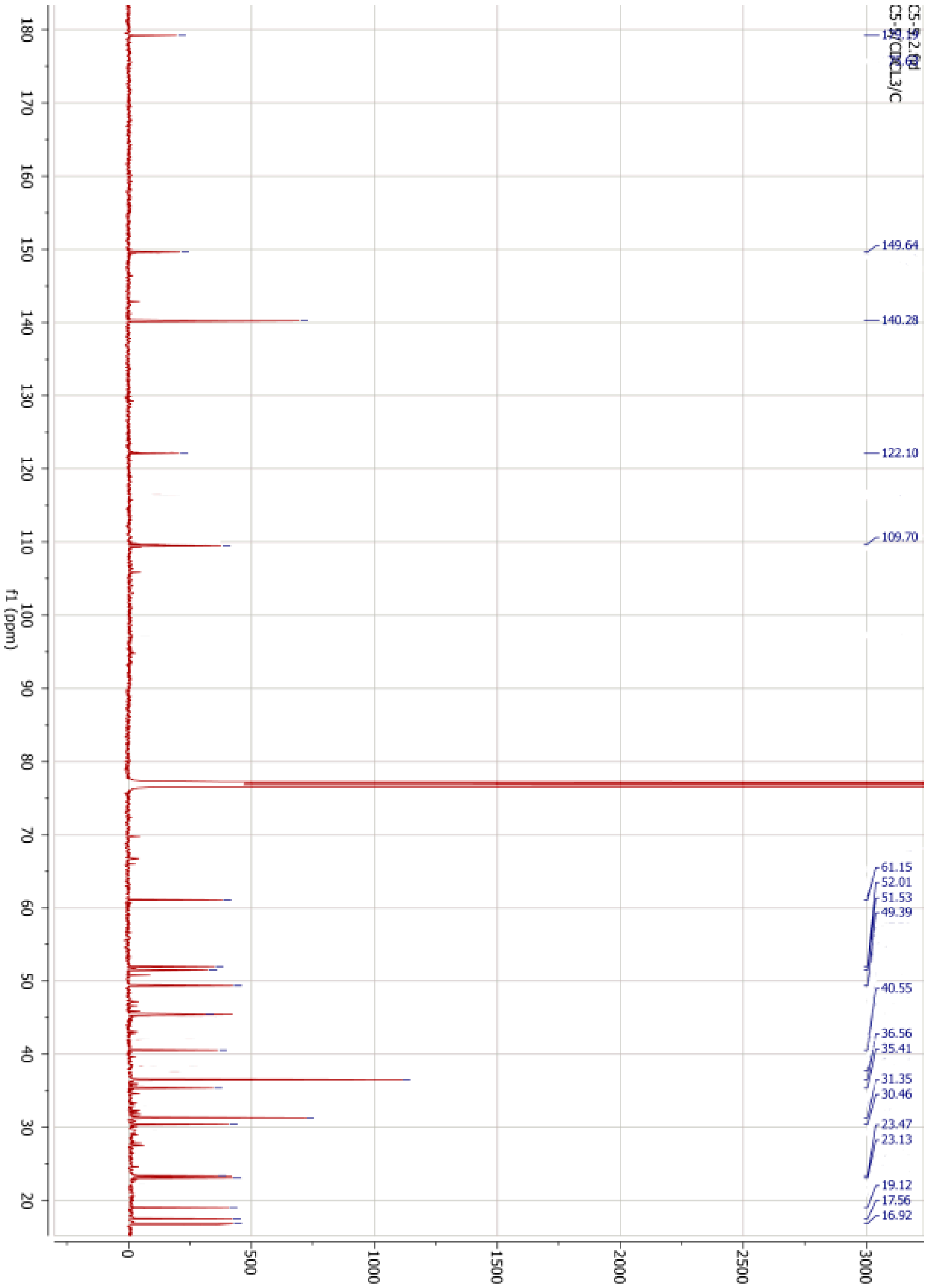

Figure S2. ^{13}C-NMR spectrum (125 MHz, $CDCl_3$) of phanginin R (**1**).

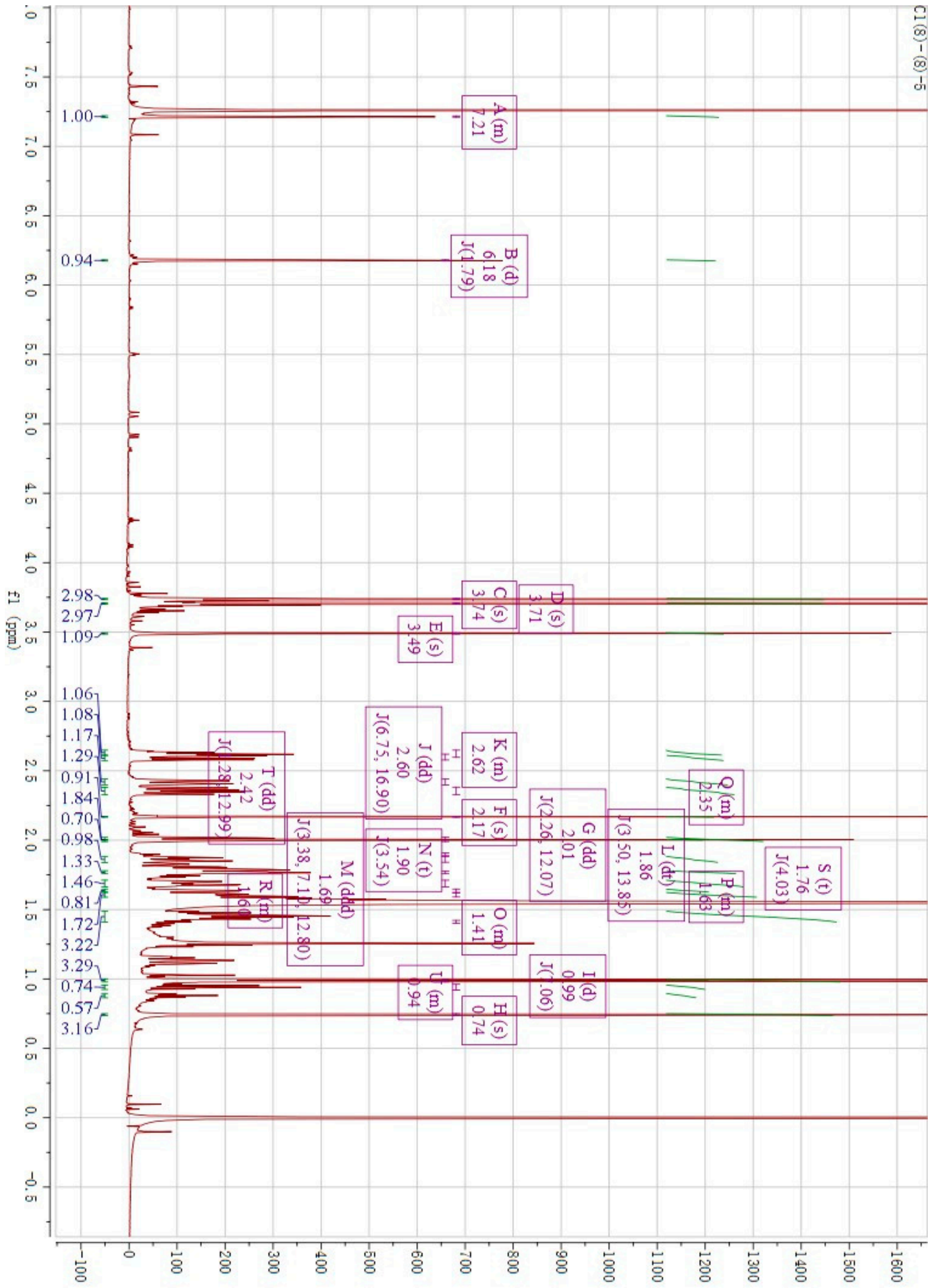

Figure S3. ^{1}H-NMR spectrum (600 MHz, $CDCl_3$) of phanginin S (**2**).

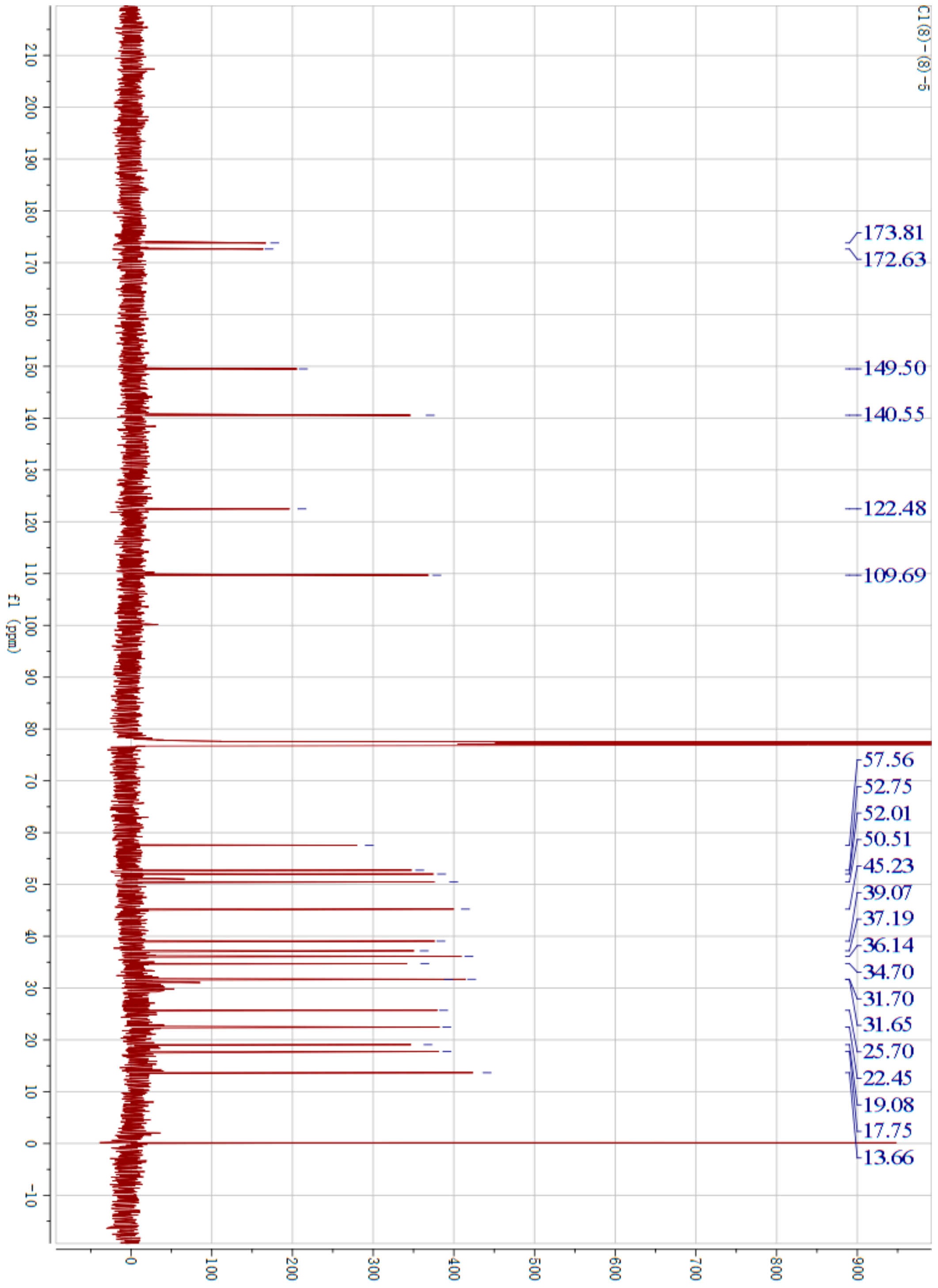

Figure S4. ^{13}C-NMR spectrum (125 MHz, $CDCl_3$) of phanginin S (**2**).

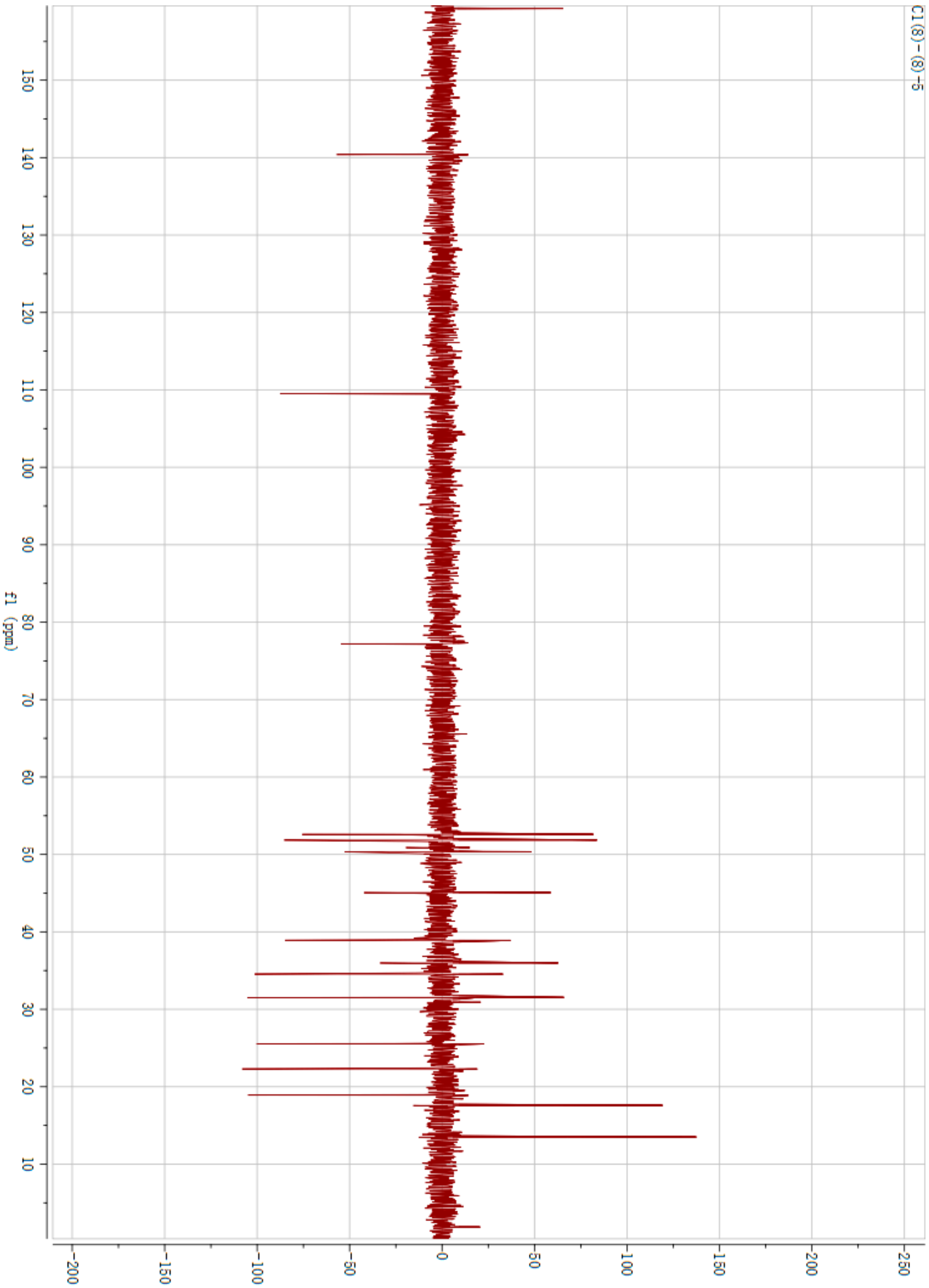

Figure S5. DEPT-135 spectrum (125 MHz, $CDCl_3$) of phanginin S (**2**).

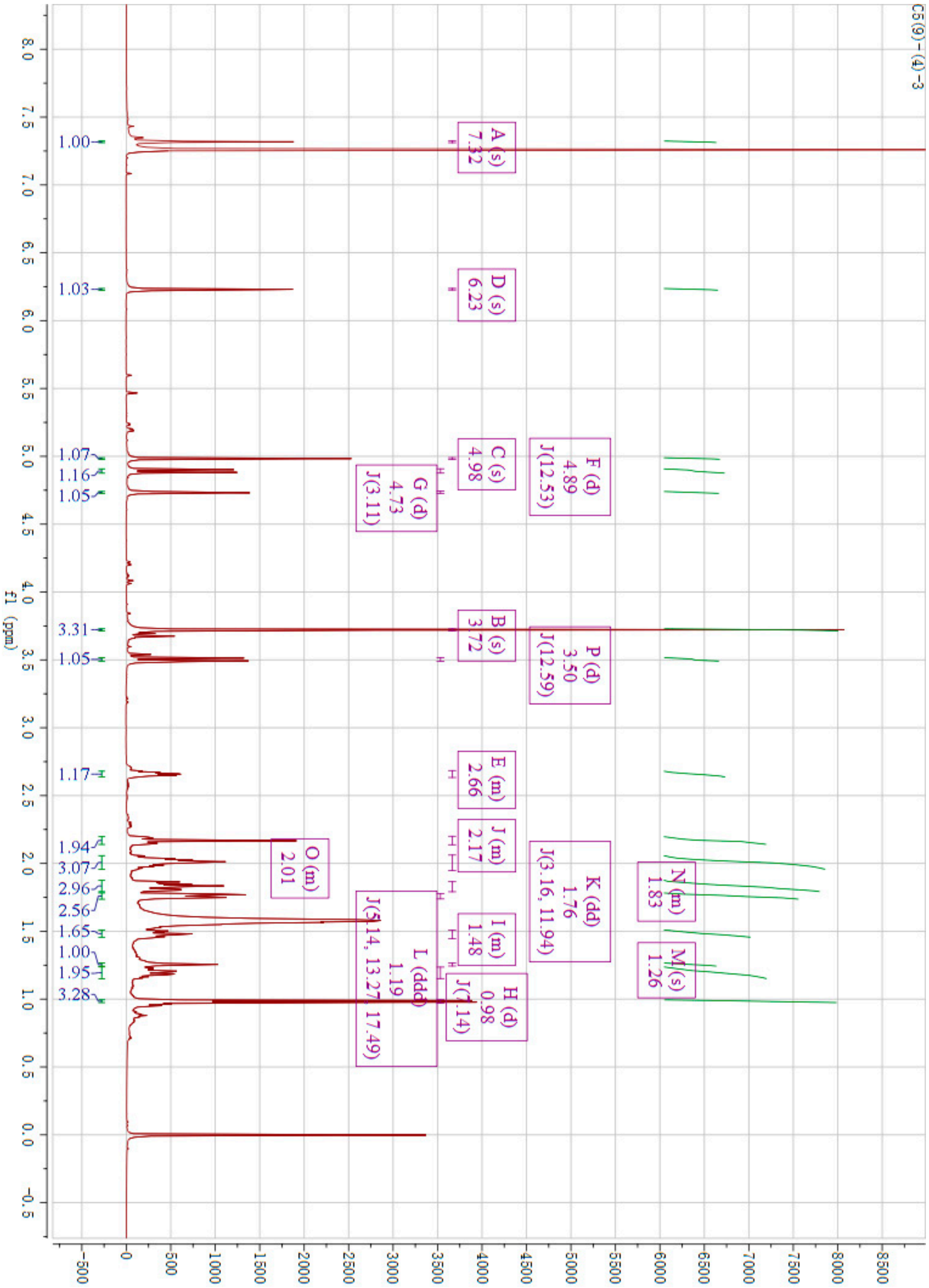

Figure S6. ^{1}H-NMR spectrum (600 MHz, $CDCl_3$) of phanginin T (**3**).

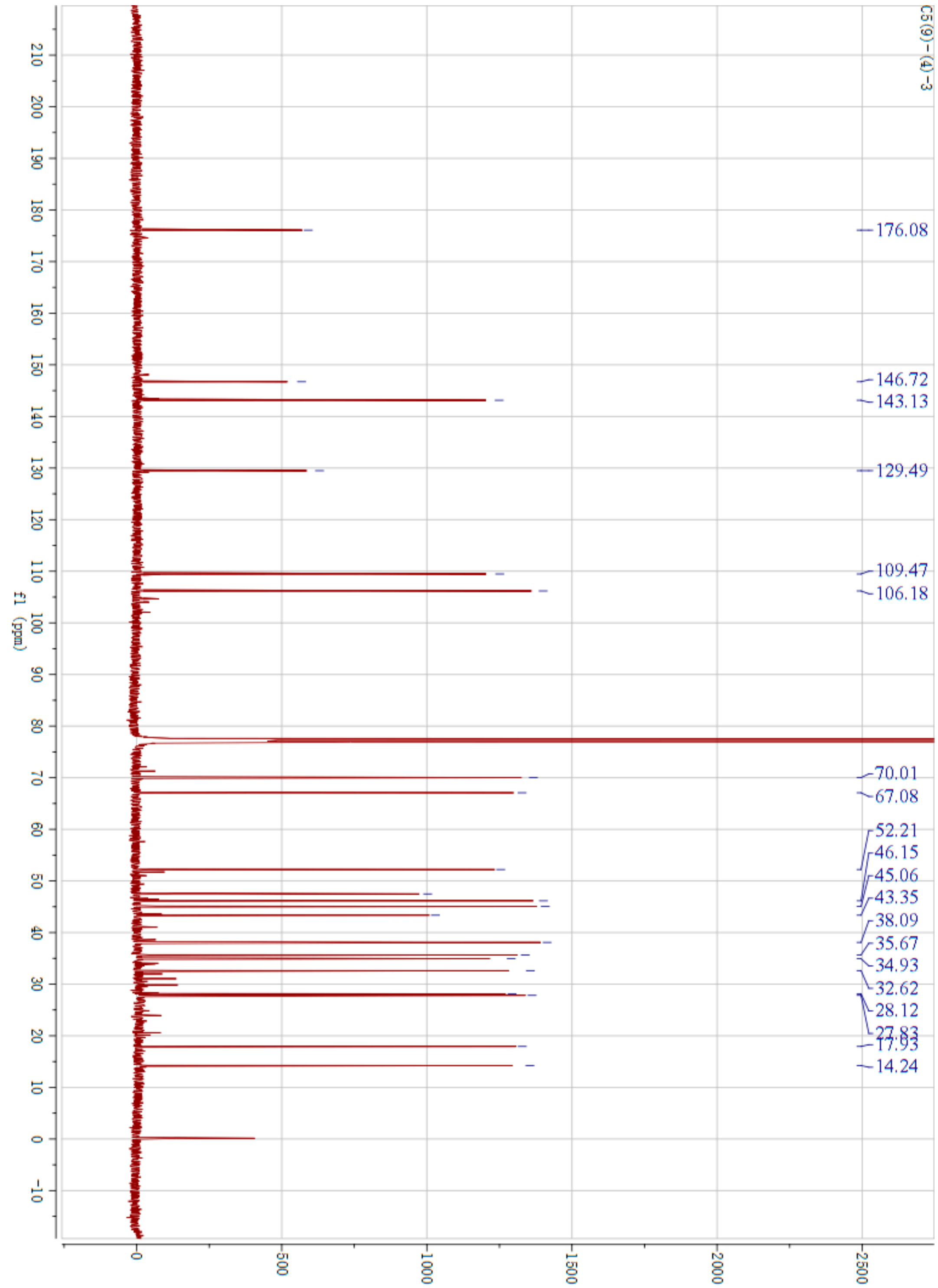

Figure S7. ^{13}C-NMR spectrum (125 MHz, $CDCl_3$) of phanginin T (**3**).

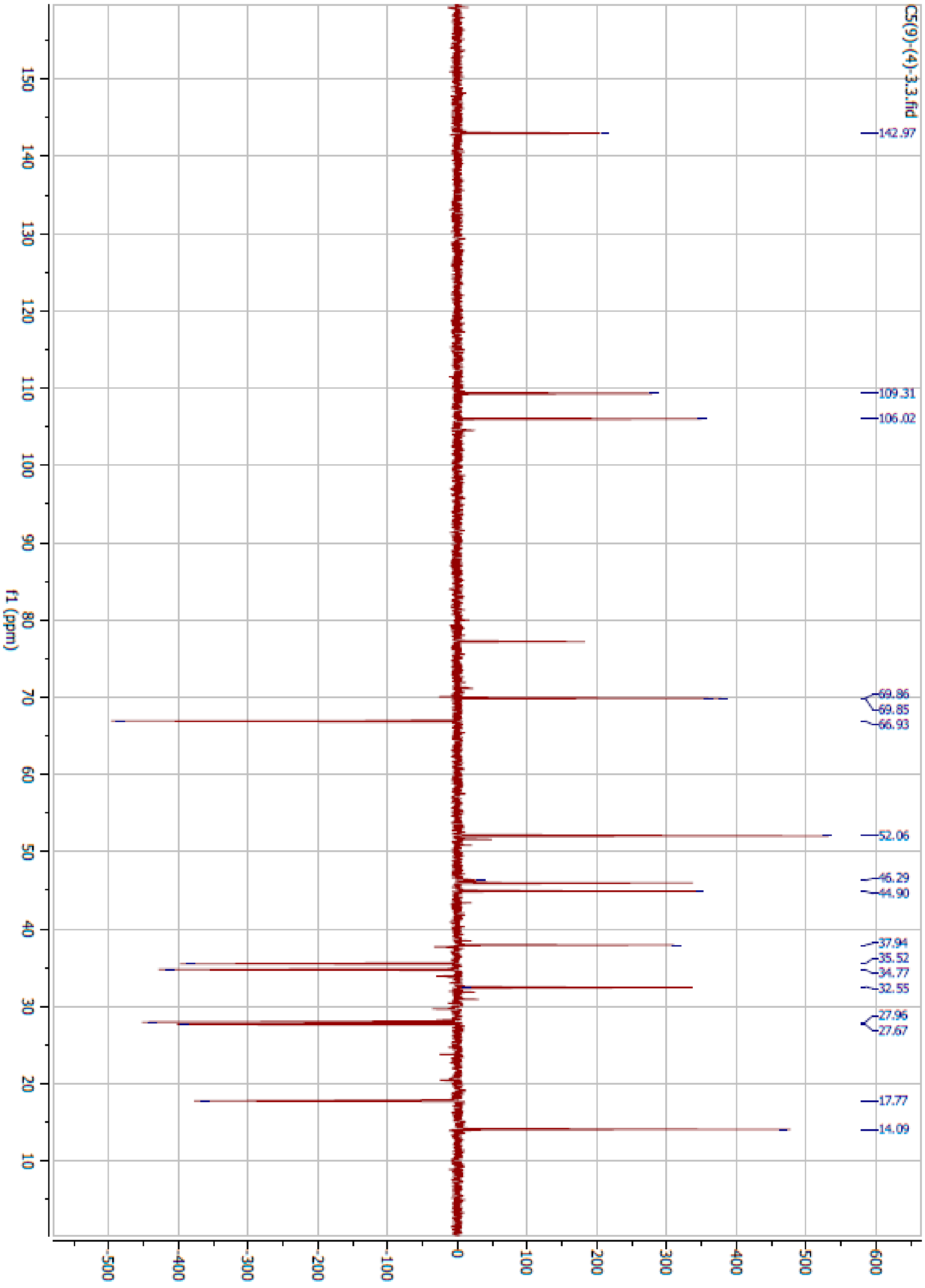

Figure S8. DEPT-135 spectrum (125 MHz, $CDCl_3$) of phanginin T (**3**).

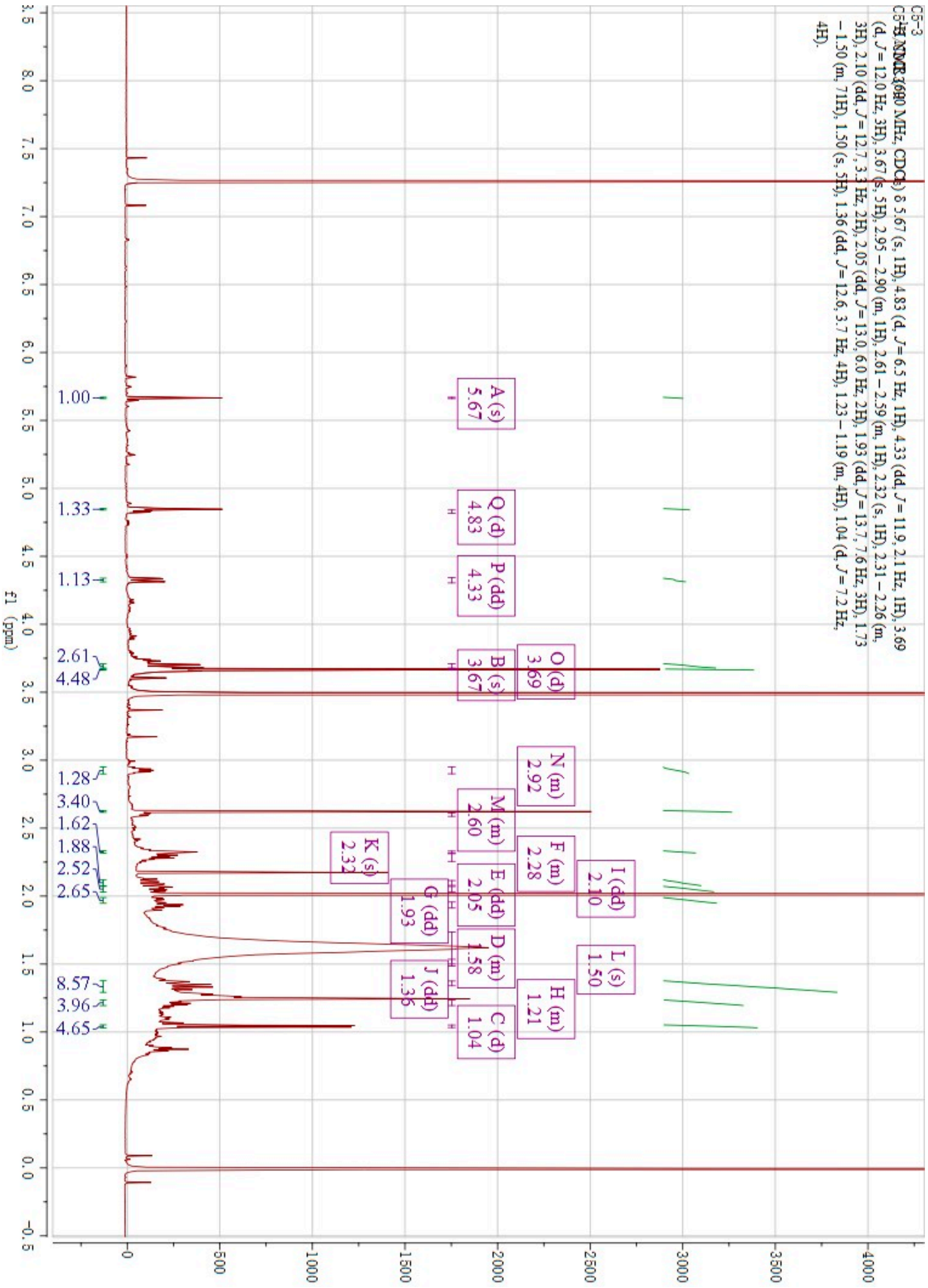

Figure S9. ^{1}H-NMR spectrum (600 MHz, $CDCl_3$) of caesalsappanin M (**4**).

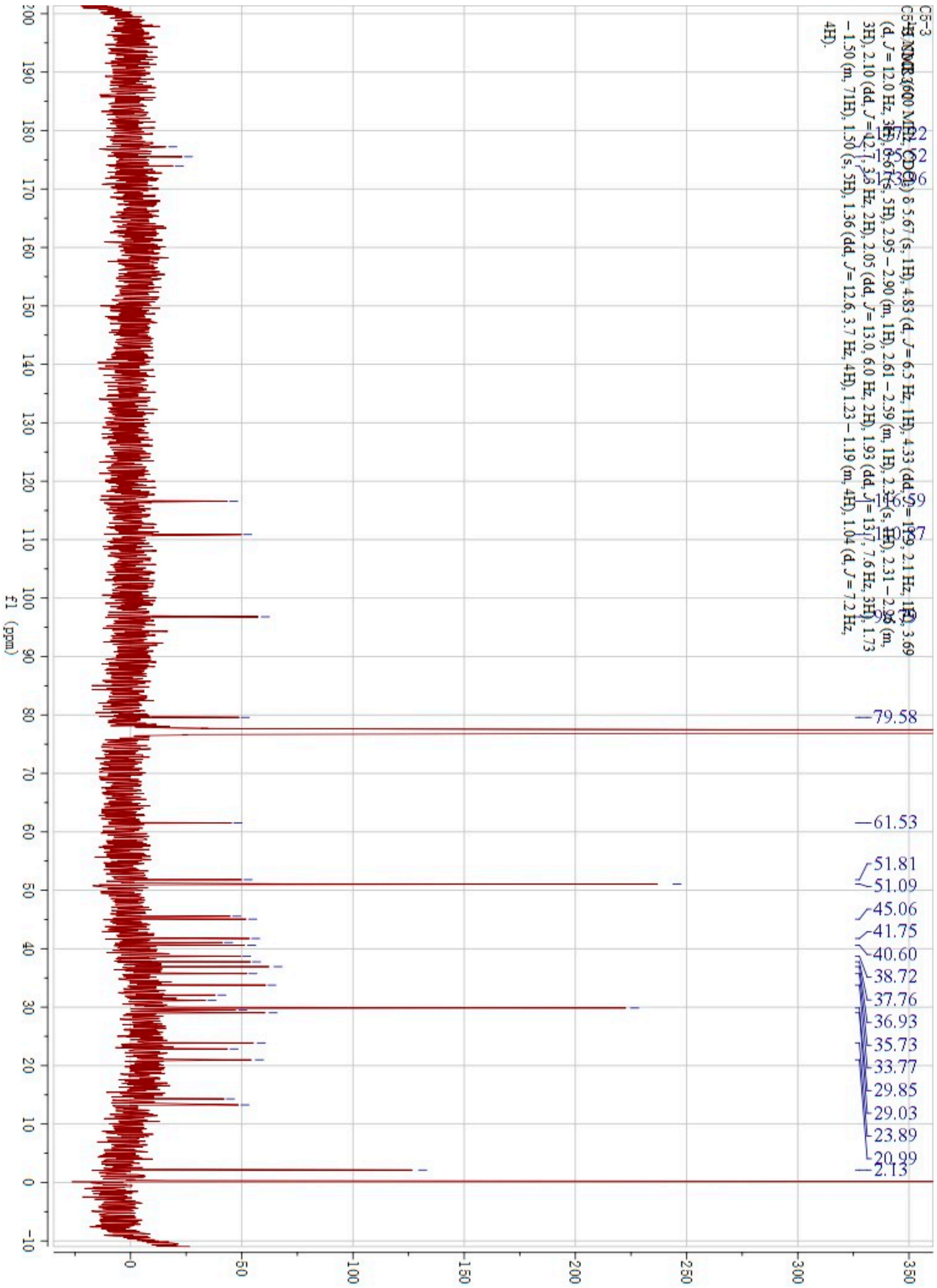

Figure S10. ^{13}C-NMR spectrum (125 MHz, $CDCl_3$) of caesalsappanin M (**4**).

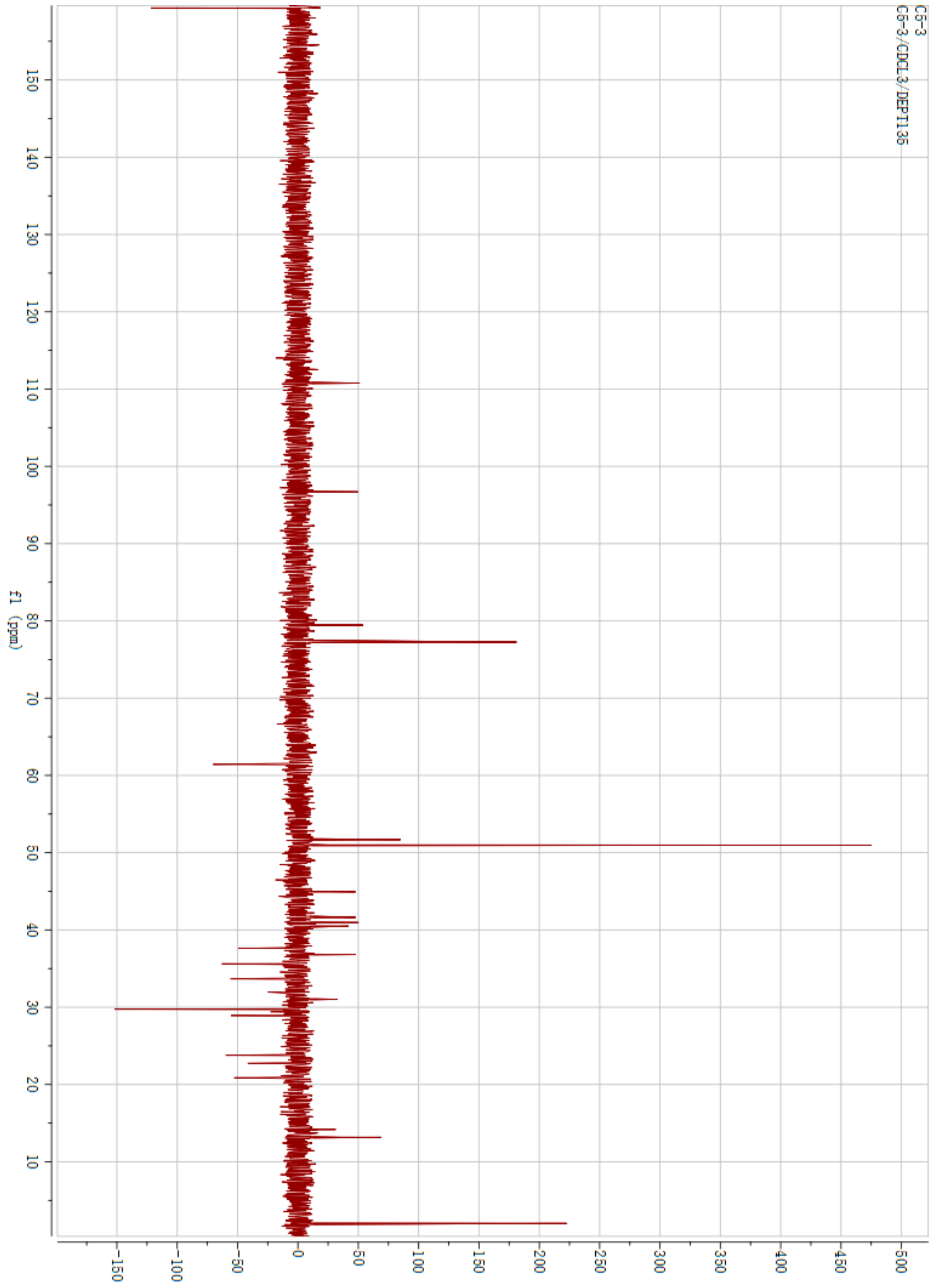

Figure S11. DEPT-135 spectrum (125 MHz, $CDCl_3$) of caesalsappanin M (**4**).

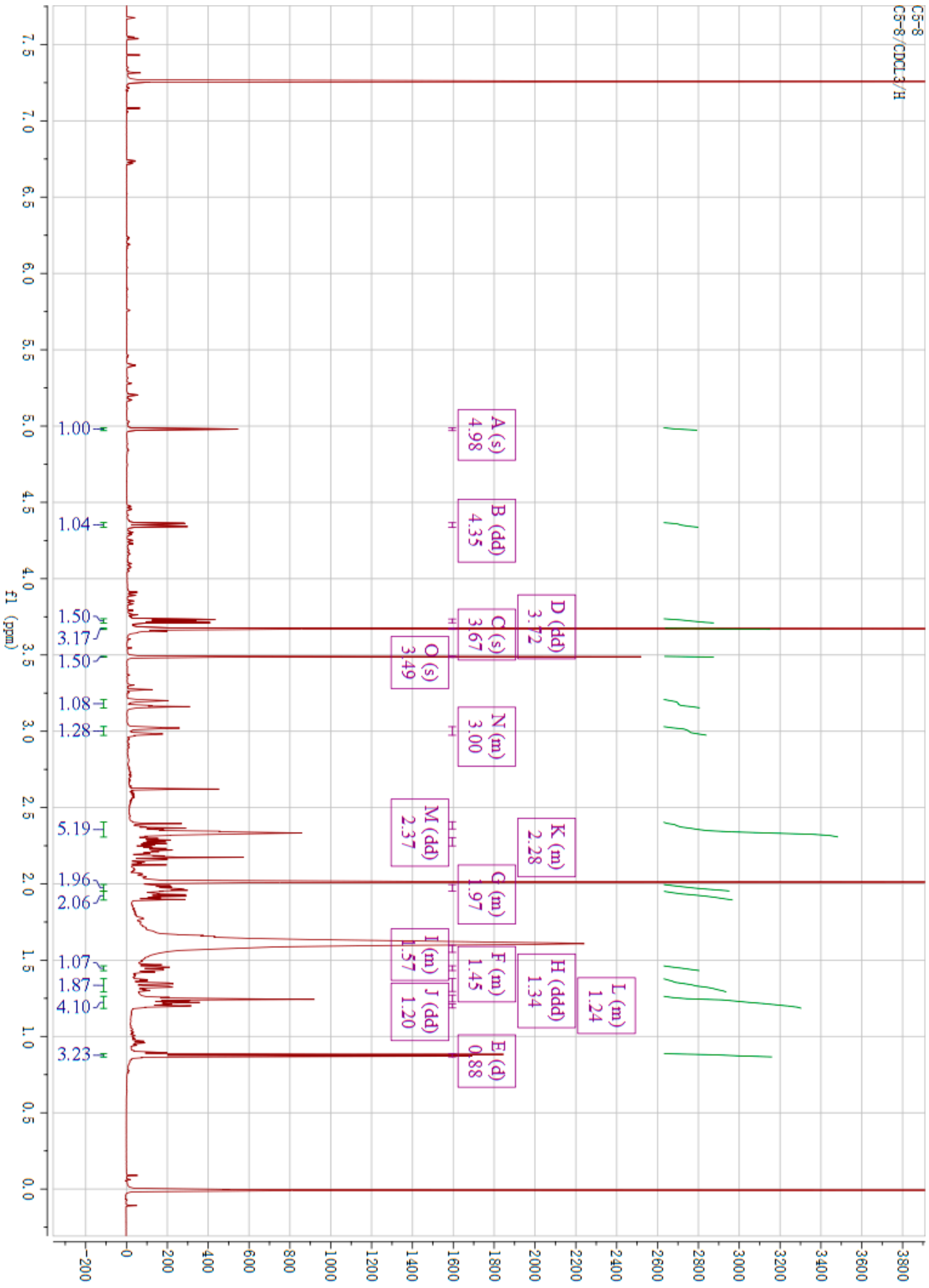

Figure S12. ^{1}H-NMR spectrum (600 MHz, $CDCl_3$) of caesalsappanin N (**5**).

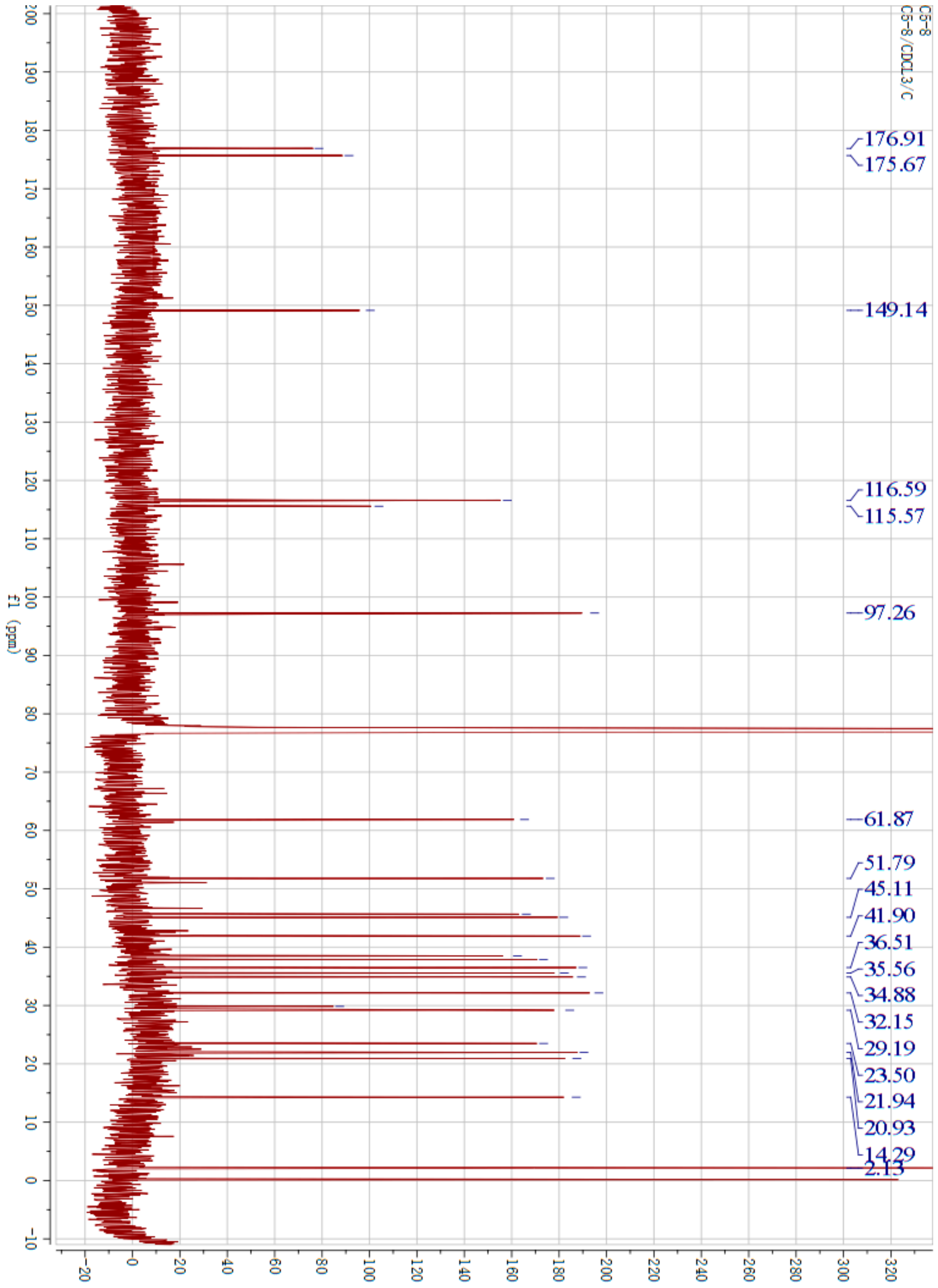

Figure S13. ^{13}C-NMR spectrum (125 MHz, $CDCl_3$) of caesalsappanin N (**5**).

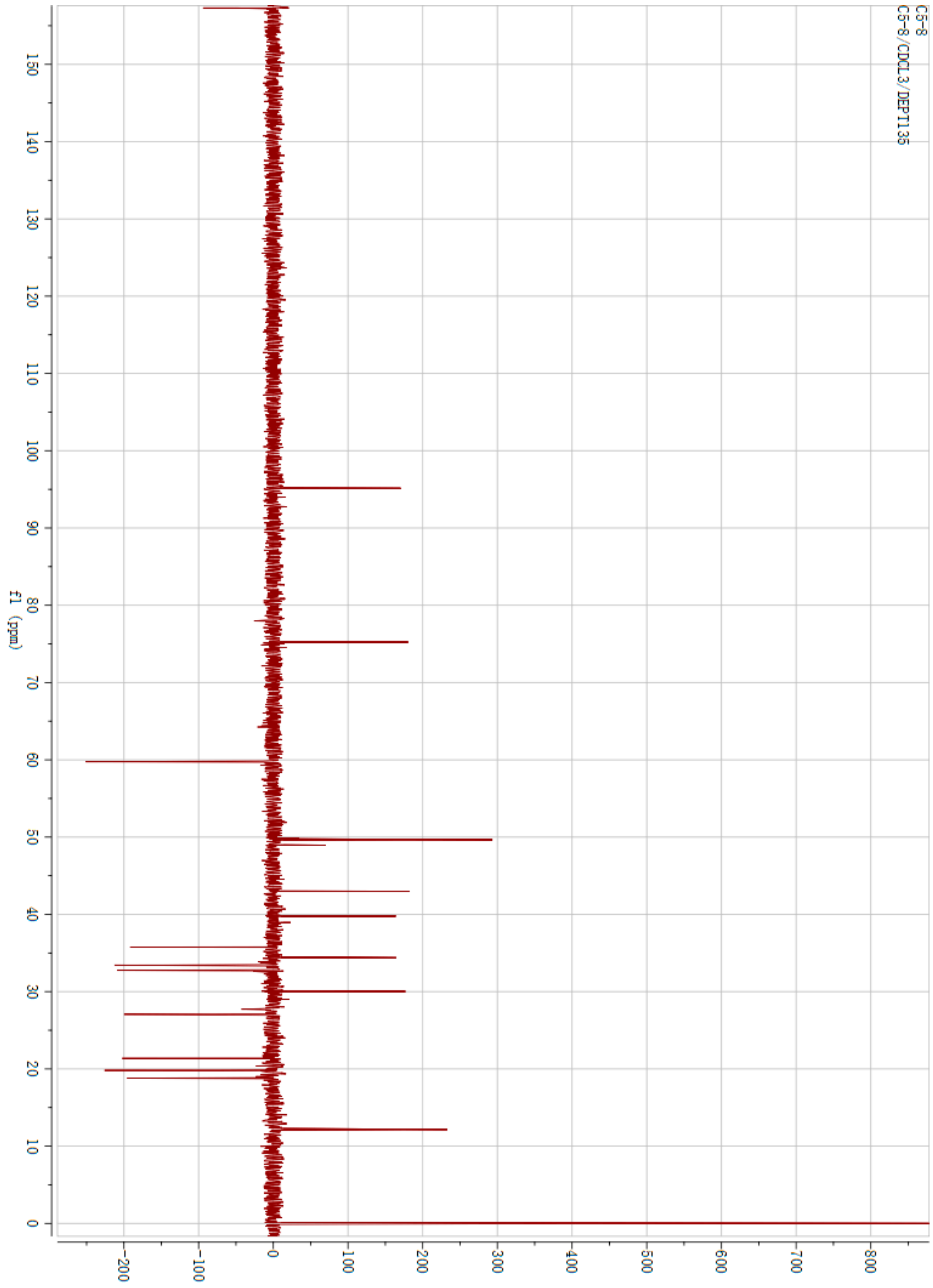

Figure S14. DEPT-135 spectrum (125 MHz, $CDCl_3$) of caesalsappanin N (**5**).

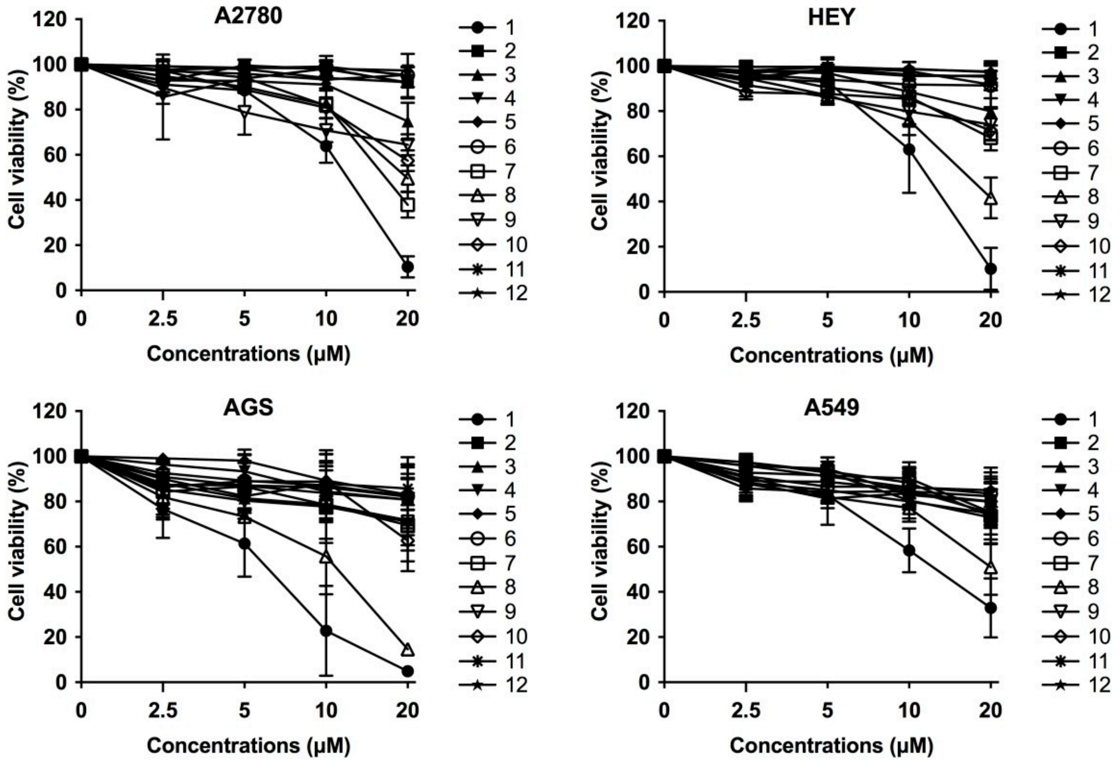

Figure S15. Concentration-response curve of A2780, HEY, AGS and A549 cells incubated with twelve compounds. Cells were treated with indicated concentrations of the compounds for 48 h and the cell viability was detected by MTT assay.
